# Orally Deliverable Nanotherapeutics for the Synergistic Treatment of Colitis-Associated Colorectal Cancer

**DOI:** 10.7150/thno.38081

**Published:** 2019-10-12

**Authors:** Weidong Han, Binbin Xie, Yiran Li, Linlin Shi, Jianqin Wan, Xiaona Chen, Hangxiang Wang

**Affiliations:** 1 Department of Medical Oncology; Sir Run Run Shaw Hospital; School of Medicine, Zhejiang University, Hangzhou, 310016, PR China.; 2 The First Affiliated Hospital; Key Laboratory of Combined Multi-Organ Transplantation, Ministry of Public Health; School of Medicine, Zhejiang University, Hangzhou, 310003, PR China.

**Keywords:** oral delivery, anti-inflammation, cytotoxic nanoparticle, self-assembly, colitis-associated colorectal cancer

## Abstract

**Purpose:** Colitis-associated colorectal cancer (CAC) poses substantial challenges for effective treatment. Currently, there is a considerable need for the development of orally bioavailable dosage forms that enable the safe and effective delivery of therapeutic drugs to local diseased lesions in the gastrointestinal tract.

**Experimental Design:** In this study, we developed orally deliverable nanotherapeutics for the synergistic treatment of inflammatory bowel diseases (IBDs) and CAC. Water-insoluble curcumin (CUR) and 7-ethyl-10-hydroxycamptothecin (SN38), which served as anti-inflammatory and cytotoxic agents, respectively, were chemically engineered into hydrophilic mucoadhesive chitosan for the generation of chitosan-drug amphiphiles.

Results: The resulting amphiphilic constructs formed core-shell nanostructures in aqueous solutions and were orally administered for *in vivo* therapeutic studies. Using a preclinical CAC mouse model, we showed that the orally delivered nanotherapeutics locally accumulated in inflamed intestinal regions and tumor tissues. Furthermore, the therapeutic synergy of the combined nanotherapeutics in CAC mice was evaluated. Compared with their individual drug forms, combined CUR and SN38 nanoparticles yielded synergistic effects to alleviate intestinal inflammation and protect mice from ulcerative colitis. Notably, the combinatorial therapy demonstrated a remarkable tumor shrinkage with only ~6% of the total tumors exceeding 4 mm in diameter, whereas ~35% of tumors were observed to exceed a diameter of 4 mm in the saline-treated CAC mice. These data suggest a new and reliable approach for improving the treatment of IBD and CAC.

**Conclusions:** Our results showed that bioadhesive chitosan materials can be used to produce colloidal-stable nanotherapeutics that are suitable for oral delivery. Both nanotherapeutics exhibited substantial accumulation in inflamed intestinal regions and tumor tissues and showed good synergy for treating CAC, warranting further clinical translation.

## Introduction

Colorectal cancer is one of the most prevalent malignancies in humans. The lifetime risk of developing colorectal cancer is 5-6% among the adult population, and nearly half of patients die from this disease 1, 2. Inflammatory bowel diseases (IBDs), such as ulcerative colitis (UC) and Crohn's disease, are chronic and debilitating inflammatory conditions 3. Depending on the duration and extent of inflammation, long-standing UC patients (~18%) have a much higher risk of developing colitis-associated colorectal cancer (CAC) than the general population 4, 5. Current clinically available therapies adopted for the management of CAC are based on various drugs, and most of them are typically administered intravenously 6, 7. Repeated intravenous injection compromises patient compliance and can also lead to potential systemic toxicity, thereby hampering long-term therapies 8. Therefore, there is a considerable need to develop practical and expedient formulations that safely and effectively deliver therapies to diseased intestinal tissues.

Oral administration of therapeutic drugs is preferred over parenteral injections and substantially improves drug safety, convenience and patient compliance 9. Moreover, the oral route facilitates local and preferential drug delivery and accumulation in the target lesions of the gastrointestinal tract while sparing healthy tissues and organs 10. For the treatment of chronic intestinal diseases (e.g., IBDs and CAC), frequent exposure of diseased intestinal sites to therapies while reducing the drug concentration in the blood is necessary. Therefore, orally bioavailable dosage forms are ideal for the management of these chronic diseases. Unfortunately, most chemotherapeutic drugs are poorly water soluble and are biopharmaceutics classification system (BCS) class II compounds 11. In addition, they are prone to loss of activity under harsh conditions of the gastrointestinal tract and ineffectively penetrate the intestinal epithelia and membranes of target cells. These factors cause poor retention in the gastrointestinal tract, insufficient stability and low and variable oral bioavailability.

Curcumin (CUR) is derived from the rhizome of *Curcuma longa* L. and has numerous pharmacological activities. The therapeutic potential of CUR has recently been shown to attenuate inflammation by effectively scavenging various reactive oxygen species (ROS) 12. Furthermore, 7-ethyl-10-hydroxycamptothecin (SN38), which is a highly potent inhibitor of DNA topoisomerase I, efficiently induces apoptosis in a wide range of cancer cells 13, 14. However, both agents are BCS class II compounds and exhibit low bioavailability when orally administered. Therefore, to use these compounds as orally available therapies, the development of therapeutically viable strategies to overcome these pharmacological limitations is necessary. Chitosan is a naturally occurring biodegradable polysaccharide 15. Considering it great abundance in nature and nontoxicity in biological systems, chitosan has been engineered to serve as a nanocarrier for drug formulations and as an effective intestinal absorption enhancer 16. Inspired by these studies, we speculated that the rational attachment of water-insoluble drugs to the side chain of chitosan derivatives could be used to create chitosan-drug amphiphiles, which are capable of recapitulating the self-assembly process in water and generating nanotherapeutics to enhance gastrointestinal absorption.

**Figure 1:**
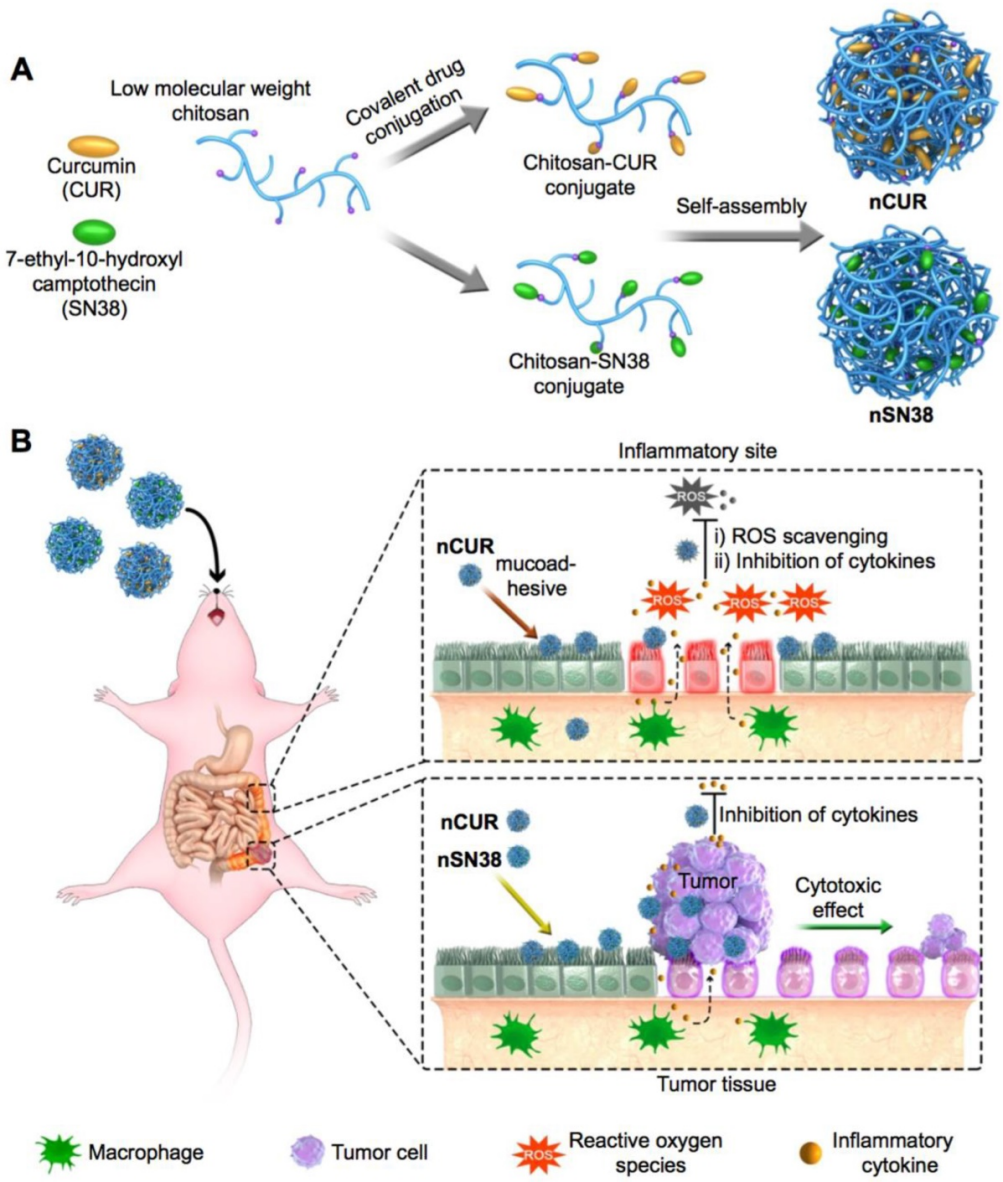
** A schematic of the self-assembly of chitosan-drug conjugates to form nanotherapeutics (i.e., nCUR and nSN38) and oral administration of the nanotherapeutics for CAC treatment.** (**A**) Therapeutic SN38 and CUR agents were individually tethered to carboxylated chitosan by a hydrolyzable linkage. The formed chitosan-drug conjugates self-assembled into stable colloidal and bioadhesive nanotherapeutics suited for oral delivery. (**B**) After oral administration by drinking water containing the therapeutics ad libitum, the **nCUR** and **nSN38** nanotherapeutics were tightly adhered to intestinal villi and efficiently accumulated in the inflamed colon tissues and tumors. Subsequently, chemically unmodified anti-inflammatory CUR and cytotoxic SN38 agents can be released to impair colitis and tumor growth, respectively.

Motivated by this rationale, we reported the design of orally deliverable nanotherapeutics for the synergistic treatment of IBD and IBD-associated colorectal cancer in mouse models in this study (**Figure 1**). Therapeutic CUR and SN38 agents were individually integrated into a chitosan scaffold by a self-hydrolyzable ester linkage (**Figure 1A**). The hydrophobicity of the drugs converted chitosan into an amphiphile construct, enabling the formation of core-shell nanostructures in aqueous media that are well suited for oral administration. Using a preclinical CAC mouse model, we showed that these nanotherapeutics are capable of substantial accumulation in inflamed intestinal tissues and tumor sites when orally administered (**Figure 1B**). Furthermore, we examined the synergy of the combined nanotherapeutics compared to their individual free drug forms to treat mice with CAC. Our results provide a design rationale for using mucus-adhering materials for the development of a nanotherapeutic that significantly accumulates in the colonic mucosa area, especially in inflammatory and tumor sites, and has an extremely high efficacy in CAC mice, warranting further clinical translation.

## Materials and Methods

For details, see Supplementary Material and Methods.

### Isolation of Murine Bone Marrow-Derived Macrophages (BMDMs)

BMDMs were obtained and cultured as previously described 17. Briefly, bone marrow cells were differentiated into BMDMs with macrophage colony-stimulating factor (M-CSF) (PeproTech, State of New Jersey, USA). After incubation for three days, half of the BMDM culture media was replaced with fresh media. On day 4, BMDMs were rinsed with Dulbecco's modified Eagle's medium (DMEM) to remove nonadherent cells, and BMDMs were used for subsequent experiments.

### Cell Lines and Culture

The mouse macrophage cell line Raw264.7 was obtained from the Cell Bank of the Chinese Academy of Science (Shanghai, China) and was used between the 8th and 10th passages. Raw264.7 cells and mouse BMDMs were cultured in DMEM supplemented with 10% heat-inactivated fetal bovine serum (Gibco, CA, USA) in a humidified 5% CO_2_ incubator at 37°C.

### Animal Experiments

All animal studies were conducted in accordance with the National Institutes of Health Guide for the Care and Use of Laboratory Animals. The experimental protocols were approved by the Ethics Committee of Sir Run Run Shaw Hospital, Zhejiang University School of Medicine.

### Establishment of the Colitis and CAC Models by AOM and DSS Treatments

Female C57BL/6 mice (6-7 weeks of age, 18-22 g, Shanghai Institute of Material Medicine, Chinese Academy of Science, China) were housed under aseptic conditions. The mice received a single intravenously injection of 10 mg/kg azoxymethane (AOM) (Sigma-Aldrich, USA). After seven days, the mice were given 3% dextran sulfate sodium (DSS) (MP Biomedical, Santa Ana, USA) in drinking water for 7 days to induce colitis. For the CAC model, the mice were further given three rounds of 2% DSS in drinking water.

### Assessment of the Targeting Ability of nCUR to Inflammatory Sites

For near-infrared (NIR) fluorescence imaging, **nCUR** was labeled with cyanine5.5 amine (Cy5.5-NH_2_, Lumiprobe, USA). Briefly, Cy5.5-NH_2_ was added to a reaction mixture containing CUR and carboxylated chitosan in the presence of 1-ethyl-3-(3-dimethylaminopropyl)carbodiimide (EDC) and 4-dimethylaminopyridine (DMAP). The molar ratio of Cy5.5 to CUR was 1:10. The reaction mixture was stirred for 24 h at room temperature. Finally, the resulting solution was dialyzed for three days in a water/methanol mixture (1:4 v/v) using a cellulose membrane (12-14,000 Da molecular weight cutoff (MWCO); Spectrum Laboratories, USA) and further dialyzed for 24 h in deionized (DI) water to yield Cy5.5-labeled **nCUR** (termed **Cy5.5-nCUR**).

The colitis model was established in female C57BL/6 mice. Healthy control mice were administered normal water only. On day 7, 100 µl of **Cy5.5-nCUR** and free Cy5.5 (both at a dose of 50 µg Cy5.5 equivalent per mouse) were administered to the mice (n = 3/arm) via oral gavage. After 5 h, the mice were sacrificed, and the major organs (colon, heart, liver, spleen, lung, and kidney) were excised. *Ex vivo* NIR fluorescence imaging was conducted using a Xenogen IVIS Spectrum* in vivo* imaging system (PerkinElmer, Waltham, MA, USA).

### CAC-Specific Accumulation of nSN38 Nanoparticles

To evaluate the tumor-targeting ability of the nanotherapeutics, we performed NIR fluorescence imaging in the CAC mouse model. For this purpose, **nSN38** nanoparticles were labeled with Cy5.5-NH_2_ to yield **Cy5.5-nSN38** using the same protocol as that for synthesizing **Cy5.5-nCUR**. One hundred microliters of **Cy5.5-nCUR** and free Cy5.5 (both at a dose of 50 µg Cy5.5 equivalent per mouse) were administered to the mice (n = 3/arm) via oral gavage. At 6 and 24 h postadministration, the mice were sacrificed, and the major organs were excised for *ex vivo* NIR fluorescence imaging.

### *In Vivo* Anti-Inflammatory Efficacy of Orally Administered nCUR

The AOM/DSS-induced colitis model established in C57BL/6 mice was used for the evaluation of anti-inflammatory activity. Mice with colitis were randomly divided into 5 groups (n = 5 or 7/arm). During the 19 days of the early stage of CAC, treatments with free CUR (50 mg/kg per day, intragastric administration) and **nCUR** (50 mg/kg per day, 0.25 mg/mL in autoclaved water administered ad libitum) were given to the CUR and **nCUR** groups, respectively. The severity of colitis is presented as the disease activity index (DAI), as previously described 18. We performed a clinical assessment of colitis by scoring body weight loss, stool consistency and blood in stool. The macroscopic scoring was performed in a single-blind manner. At day 19, the mice were euthanized, and the distance between the ileocecal junction and proximal rectum was measured.

### *In Vivo* Antitumor Efficacy

The therapeutic efficacy of the combination of **nCUR** and **nSN38** was evaluated in the CAC mouse model. To establish the CAC model, C57BL/6 mice were intravenously injected with AOM (10 mg/kg) followed by ad libitum administration of 2% DSS in water for three cycles. At day seven postadministration of AOM, the mice were randomly divided into five groups (n = 12/arm). The mice were treated with CPT-11, CUR plus CPT-11, and **nCUR**/**nSN38**. The therapies using CUR (50 mg/kg per day, intragastric administration) and **nCUR** (50 mg/kg per day, 0.25 mg/mL in water administered ad libitum) were initiated on the day seven. Furthermore, at day 70, the treatments using **nSN38** (20 mg/kg per day, 0.1 mg/mL in water administered ad libitum) and intraperitoneal injection of CPT-11 at a dose of 42 mg/kg for five times per week (equivalent to 20 mg/kg of SN38 per day) were initiated. Anticancer SN38 treatment continued for four weeks. The mice were euthanized at day 98, and tumor numbers and sizes were measured. The length (*L*) and width (*W*) of tumors were measured with calipers and tumor volume was calculated using the following formula: *V* = (*L* × *W*2)/2, *W* being smaller than *L*. The tumor growth inhibition (TGI) was evaluated by calculating the total tumor volume (*TV*) of mice in different treatment groups using the following formula: TGI = (1 - *TV*_treatment group_/*TV*_untreated group_) × 100%.

### Statistical Analysis

Statistical analysis was performed by a two-tailed Student's t-test, one-way and two-way ANOVA followed by Bonferroni's post hoc test, and log-rank test. *p* < 0.05 indicates statistical significance (*), *p* < 0.01 indicates high significance (**), and *p* < 0.001 indicates very high significance (***). All data are presented as the mean ± standard deviation (SD).

## Results

### Rational Design and Self-Assembly of the Orally Deliverable Nanotherapeutics

Previous studies demonstrated that intestinal absorption of chitosans *via* oral administration highly relies on the molecular weight 19, 20. Deacetylated low-molecular-weight chitosans (i.e., molecular weight (Mw) 3,000 Da) were able to quickly and reversibly open tight junctions, facilitating the transportation of conjugated drug payloads across intestinal epithelial cells. Thus, we chose commercially available chitosan as a scaffold to covalently tether multiple molecules of therapeutic agents. To achieve this goal, a two-step reaction was employed (**Synthetic scheme S1-S3**). A Michael reaction of the amine moiety with acrylic acid yielded an *N*-carboxyethyl group in the chitosan backbone 21, which further allowed the attachment of the CUR and SN38 molecules by esterification in dimethyl sulfoxide (DMSO) (the synthetic details are included in the Supplementary Material). Successful drug ligation was confirmed by ^1^H NMR analysis (**Figure S1-S3**), and the disappearance of the peaks derived from the parent drugs was verified by high-performance liquid chromatography (HPLC) analysis.

Gradual dialysis against media (a methanol-H_2_O solvent mixture) was carried out to remove excess unreacted drugs. During this process, we also hypothesized that the hydrophobicity of the attached CUR and SN38 molecules promotes the self-assembly of chitosan-drug conjugates into colloidal-stable nanoparticles. Transmission electron microscopy (TEM) images showed the formation of spherical nanostructures for the CUR- and SN38-chitosan conjugates (termed **nCUR** and **nSN38**, respectively, **Figure 1A**) upon self-assembly. The particle sizes were 44.86 ± 9.04 and 51.71 ± 15.11 nm for **nCUR** and **nSN38**, respectively, in TEM observation (**Figure S4**). Dynamic light scattering (DLS) analysis revealed that hydrodynamic diameters (*D*_H_) of **nCUR** and **nSN38** were 81.2 ± 8.0 and 118.1 ± 12.7 nm, respectively, in DI water (**Figure S4**). The larger *D*_H_ values than the particle sizes observed in the dry TEM images could be attributable to the low-contrast flexible chitosan shell layer on the particle surface and the swelling effect in water.

We further examined the release profiles of drug payloads from their covalently attached scaffolds^22^. For this purpose, the solutions such as simulated gastric fluid (SGF, pH 1.2) and simulated intestinal fluid (SIF, pH 6.8) were prepared as releasing media to simulate gastrointestinal tract. Dialysis against PBS (pH 7.4) or SIF accelerated the liberation of the drugs from the corresponding nanoparticles (**Figure S5**). In contrast, slow drug release profiles were observed against SGF with an acidic pH value; that is, only ~19.4% and ~14.3% of CUR and SN38 agents were released from **nCUR** and **nSN38**, respectively, after 144-h incubation. The distinct release behavior could be attributed to the ester linkage used for drug conjugation, which ensures the specific liberation of therapeutic agents at the targeted sites. On the other hand, the stability of the nanoparticles under harsh acidic conditions is crucial for their persistence in the gastrointestinal tract upon oral administration. Analyses of particle sizes and zeta potentials using DLS measurement evidenced that these nanotherapeutics were stable in SGF and SIF over a 24-h period (**Figure S6**). In addition, we did not observe precipitates for both nanoparticle preparations when they were stored in DI water over a long period of time (i.e., several weeks), suggesting that the nanoparticle solutions are applicable for administration in drinking water by ad libitum consumption in the subsequent animal experiments.

### nCUR Shows Anti-Inflammatory Activity and Suppresses Inflammatory Cytokines in Macrophages

**Figure 2:**
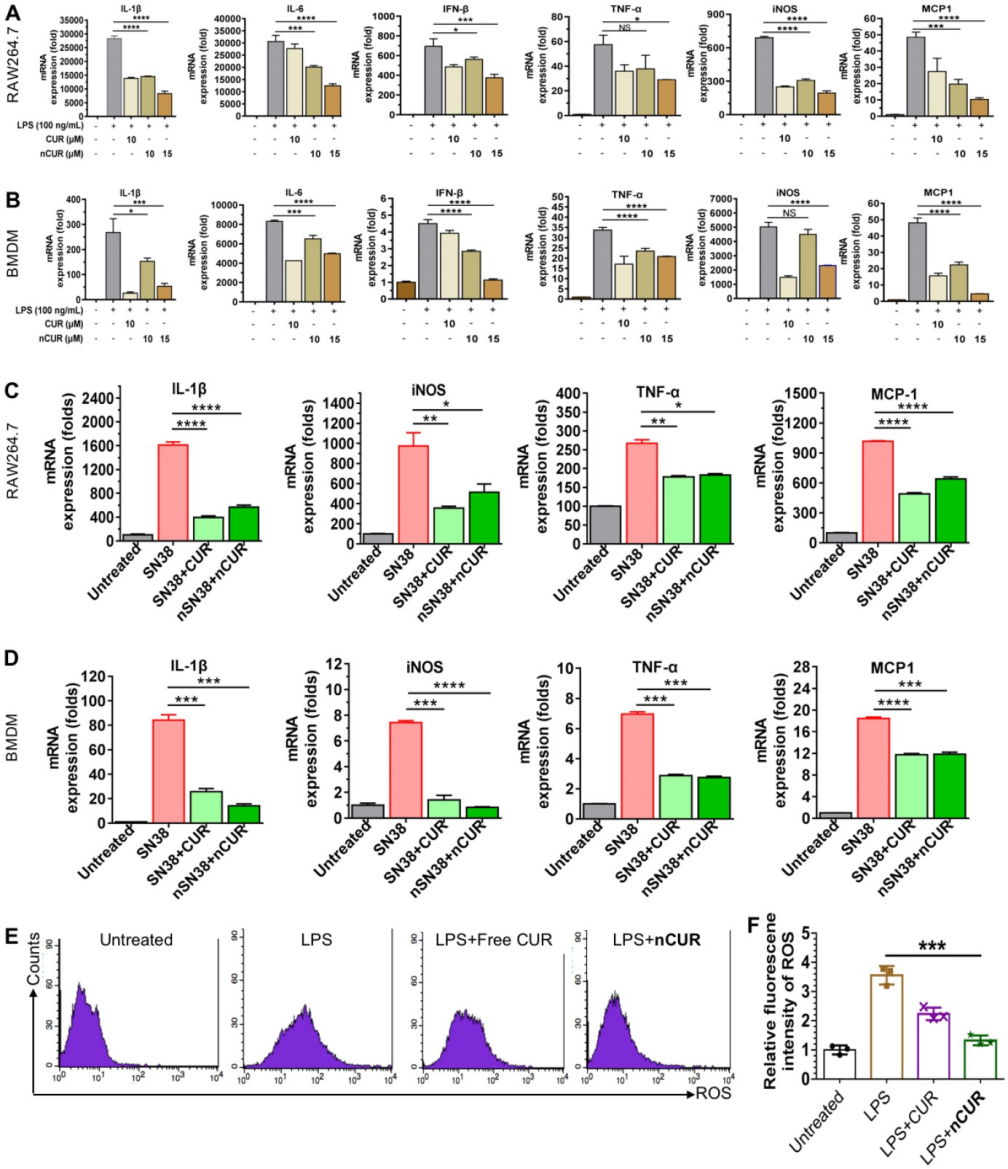
** Self-assembled nCUR inhibits inflammatory cytokines in macrophages.** (**A** and **B**) mRNA expression levels of proinflammatory cytokines were assessed when Raw264.7 cells (**A**) and bone marrow-derived macrophages (BMDMs) (**B**) were incubated with CUR and **nCUR** for 12 h followed by exposure to LPS for 3 h. (**C** and **D**) mRNA expression levels of IL-1β, TNF-α, iNOS, and MCP-1 were assessed when Raw264.7 cells (**C**) and BMDMs (**D**) were treated with vehicle, SN38, SN38 combined with CUR and SN38 combined with **nCUR** for 6 h. The data were normalized to GAPDH and then presented as values relative to the control values. (**E**) Raw264.7 cells were stimulated with LPS for 12 h in the presence of CUR and **nCUR**. Three independent staining tests were performed, and the data are presented as the mean ± SD. N.S. indicates no significant difference; *p < 0.05, **p < 0.01, and ***p < 0.001, as determined by two-way ANOVA followed by Bonferroni's post hoc test.

Previous studies have suggested that CUR has potential anti-inflammatory activity 23, 24. Furthermore, lipopolysaccharide (LPS) promotes macrophage polarization. During this process, several cytokines, such as IL-1β, IL-6, IFN-β, TNF-α, iNOS, and MCP-1, are secreted through the activation of classic inflammatory signaling pathways 25. Thus, we first used macrophages to validate whether nanoparticles assembled from CUR-tethered chitosan inhibit the secretion of inflammatory cytokines *in vitro*. Therefore, the mouse macrophage cell line Raw264.7 and primary mouse BMDMs were preincubated with **nCUR** for 12 h, followed by stimulation with LPS (500 ng/mL) for an additional 6 h. Subsequently, the total mRNA was extracted, and the expression of cytokines was quantified by real-time quantitative reverse transcriptase polymerase chain reaction (qRT-PCR). In LPS-stimulated Raw264.7 cells and BMDMs, the expression levels of IL-1β, IL-6, IFN-β, TNF-α, iNOS, and MCP-1 increased substantially (**Figure 2A**-**B**). Conversely, the addition of **nCUR** suppressed the expression of these cytokines in a dose-dependent manner. Therefore, we confirmed that **nCUR** decreases the expression levels of cytokines with an ability that is comparable to that of free CUR irrespective of covalent conjugation and nanoparticle assembly.

CPT-11 (irinotecan) is a standard-of-care chemotherapeutic for the treatment of colorectal cancer in the clinic. However, this drug causes severe side effects, such as severe diarrhea and colitis, limiting dose intensification 8, 26. Treatment with SN38, which is the active metabolite of CPT-11, has been reported to produce inflammatory cytokines 27, 28. To examine whether **nCUR** shows anti-inflammatory activity, we used Raw264.7 cells and BMDMs in this experiment. Upon treatment with SN38 (2 μM) in the presence or absence of **nCUR** (10 μM), qRT-PCR was performed to quantify the mRNA expression levels of several key cytokines. As shown in **Figure 2C**-**D**, treatment with SN38 substantially enhanced the secretion of inflammatory cytokines, including IL-1β, iNOS, TNF-α, and MCP-1, consistent with previous reports. Interestingly, we found that free CUR and **nCUR** potently reversed the upregulation of cytokines in both cell types. Thus, it is reasonable to conclude that **nCUR** inhibits proinflammatory cytokine secretion and inflammation *in vitro*.

In the initial stages of CAC, ROS, such as superoxide anion (O_2_^·-^), hydrogen peroxide (H_2_O_2_), and the hydroxyl radical (OH^·-^), are overproduced. ROS are typically involved in signal transduction and genomic instability. In addition, the overproduction of ROS is a characteristic of inflammation and CAC pathogenesis 29, 30. To investigate whether **nCUR** plays a role in scavenging ROS, Raw264.7 cells were incubated with 2',7'-dichlorofluorescin diacetate (DCFH-DA, a commercially available ROS sensor) and then analyzed by flow cytometry. As shown in **Figure 2E**-**F**, stimulation with LPS led to an increase in the fluorescence intensity of Raw264.7 cells, which was nearly 3.5 times greater than that of the untreated cells. Upon treatment with CUR and **nCUR**, the levels of intracellular ROS were reduced, indicating the potential of **nCUR** to reverse the upregulation of ROS induced by LPS stimulation in Raw264.7 cells.

### nSN38 Shows Potent Anti-proliferation Activity and Promotes Apoptosis in Colon Cancer Cell

We further examined the anti-proliferation of **nSN38** using the EdU incorporation assay in human colorectal DLD1 and HCT-116 cancer cell lines. In this experimental setting, CPT-11 and free SN38 administered in DMSO were included as controls. Treatment with **nSN38** for 48 h was observed to induce the potent inhibition of proliferation in both tested cell lines, and the inhibition rate was comparable with that of free SN38 but was remarkably higher than that of CPT-11 treatment (**Figure S7**). To validate whether the anti-proliferation effect was a consequence of apoptosis induced by the SN38 agent, we performed the Alexa Fluor 488 Annexin V/PI assay. Upon treatment with **nSN38**, a high level of early and late apoptosis was observed, showing ~57% and ~41% of the cell populations at apoptotic stages in DLD1 and HCT116 cells, respectively (**Figure S8**). This activity was higher than that of CPT-11 (i.e., 24% and 25% of apoptotic rates in DLD1 and HCT116 cells, respectively). These results suggest that covalent conjugation did not deter the cytotoxic potency and **nSN38** is able to liberate therapeutically active compound once internalized by cancer cells.

### The nCUR Nanotherapeutic Preferentially Accumulates in Inflamed Intestinal Tissue in Mice with Colitis

**Figure 3:**
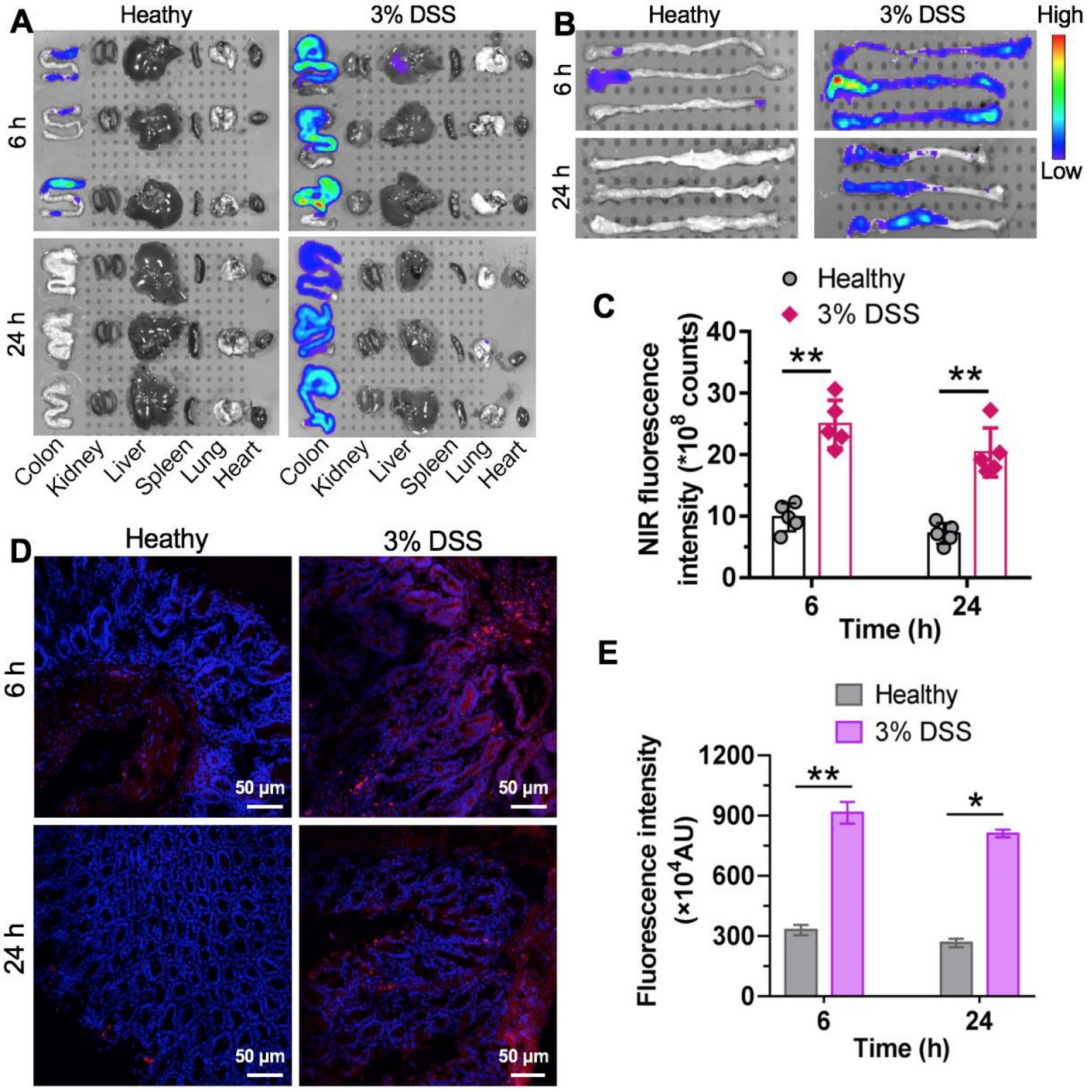
** Specific accumulation of nCUR nanotherapeutics in inflamed colons.** (**A**) Representative *ex vivo* fluorescence images for the evaluation of nanoparticle distribution. C57BL/6 mice were given drinking water containing 3% DSS ad libitum to induce intestinal inflammation for 7 days. Mice with colitis were orally administered Cy5.5-labeled **nCUR** (termed **Cy5.5-nCUR**), and healthy mice were also included as controls. At 6 and 24 h postadministration, the major organs were excised for *ex vivo* NIR imaging. (**B** and **C**) *Ex vivo* imaging (**B**) and quantification of fluorescence intensities (**C**) of colons excised from each group of mice. Confocal microscopy images (**D**) and fluorescence intensities (**E**) of colorectal tissue sections harvested at 6 and 24 h after oral administration. The red and blue signals indicate Cy5.5 and nuclei counterstained with DAPI, respectively. The data are presented as the mean ± SD. *p < 0.05, **p <0.01, and ***p < 0.001, as determined by two-way ANOVAfollowed by Bonferroni's post hoc test.

During early CAC tumorigenesis, ROS are overproduced and act as mediators of inflammation. Nanotherapeutics are expected to preferentially accumulate at inflammatory sites where vascular structures are leaky. We hypothesized that nCUR nanoparticles accumulate in inflamed colons in a colitis model after oral administration. To test this hypothesis, intestinal inflammation in C57BL/6 mice was established by ad libitum administration of 3% DSS in drinking water for 7 days. In this study, we used near-infrared (NIR) fluorescence imaging to assess the biodistribution of the nanotherapeutics in animals. This is a noninvasive technique with high sensitivity and enables real-time and quantitative analysis of NIR dye-labeled nanotherapeutics 31. Therefore, we covalently tethered the amine-bearing NIR dye Cy5.5 to the excess carboxyl group of the chitosan-CUR conjugates, yielding Cy5.5-labeled nCUR (termed Cy5.5-nCUR). After oral administration of Cy5.5-nCUR, the major organs were excised and the fluorescence intensities in each organ were quantitatively analyzed at the timepoints of 6 and 24 h (**Figure 3A** and **Figure S9**). *Ex vivo* NIR imaging revealed that the signals derived from Cy5.5-nCUR primarily accumulated in colons, while the other organs showed negligible fluorescence. More interestingly, stronger signals were observed in the colons of mice with colitis than in healthy mouse colons at both timepoints (**Figure 3B**-**C**).

To further confirm whether **nCUR** nanoparticles are deposited throughout inflamed colon tissue, frozen colon sections were randomly selected and evaluated by confocal microscopy. Fluorescence signals from the inflamed colon tissues were intense and largely persisted, even after 24 h, while the healthy controls did not show an NIR signal postadministration (**Figure 3D**-**E**). These results indicated that the **nCUR** nanotherapeutic is capable of accumulating at inflamed sites after oral administration, presumably due to the mucoadhesiveness of the nanoparticles and the enhanced vascular permeability in inflamed colon tissues.

### nCUR Enhances the Survival Rate and Alleviates Colitis in Mice

**Figure 4:**
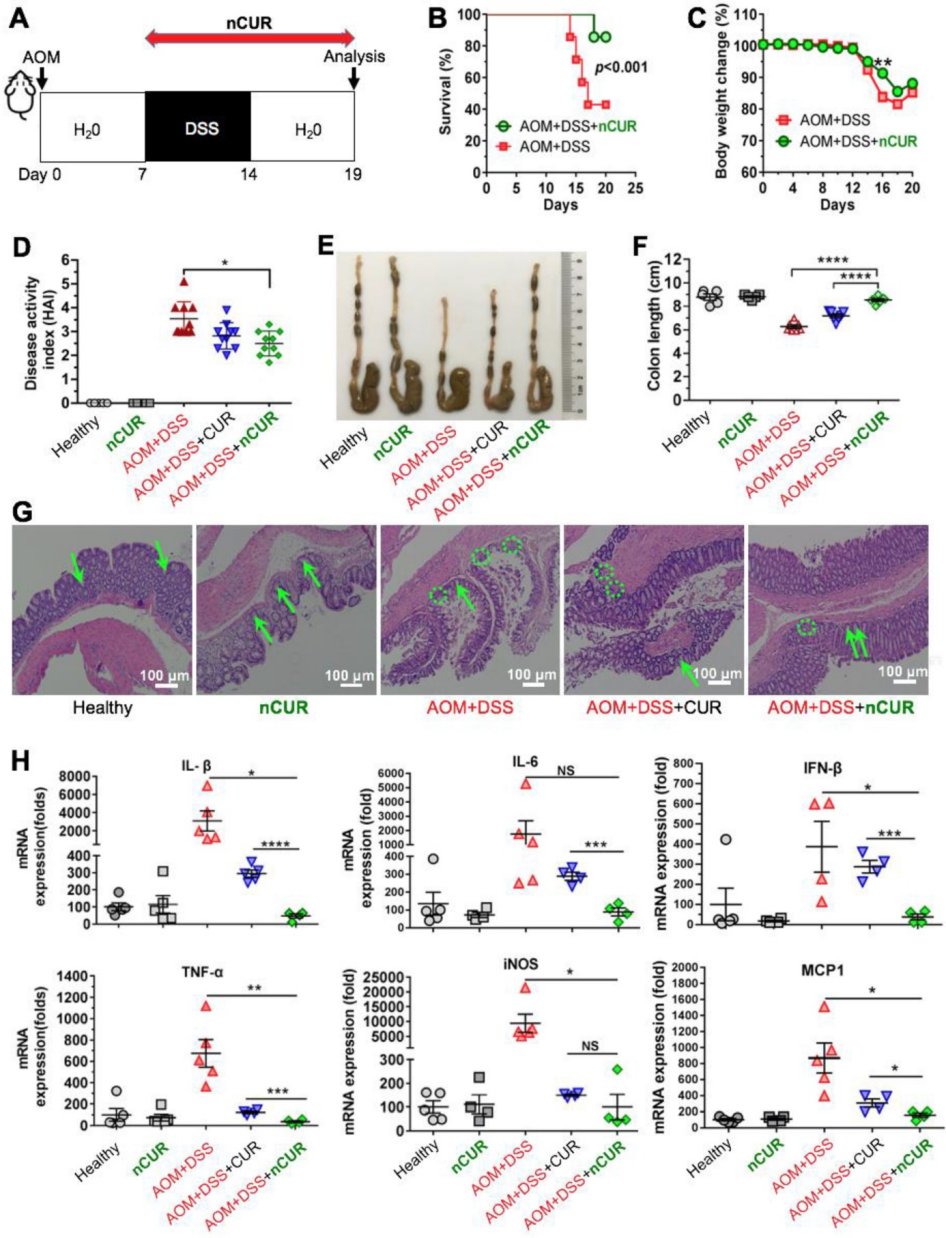
** Treatment with nCUR alleviates colitis at the early stage of CAC.** (**A**) A schematic overview of the DSS-induced colitis model in C57BL/6 mice. The mice were injected with AOM followed by supplementation with 3% DSS in water for seven days. For anti-inflammatory treatment, **nCUR** was given daily in water by drinking ad libitum. The mice were euthanized on day 19 after AOM injection. (**B**) Treatment with **nCUR** improved survival in mice with DSS-induced fatal colitis (n = 7 mice in each group). Kaplan-Meier survival curves were compared by the log-rank test. (**C**) Weight loss was monitored throughout the therapeutic studies. (**D**) The disease activity index (DAI) was evaluated on day 19 after AOM injection. DAI is the summation of the stool consistency index, fecal bleeding index, and weight loss index. (**E**) Colons were photographed, and (**F**) colon lengths were measured at the end of the therapeutic studies. (**G**) Representative H&E staining of the mouse colon. The green arrows and dotted circles indicate crypts and leukocyte infiltration, respectively. (**H**) Relative expression of inflammatory cytokines in the murine colon on day 19 after the induction of colitis in mice. The data are presented as the mean ± SD. N.S. indicates no significant difference; *p < 0.05, **p < 0.01, and ***p < 0.001, as determined by two-way ANOVA followed by Bonferroni's post hoc test.

Encouraged by the *in vitro* anti-inflammatory activity and inflammation-specific accumulation, we hypothesized that orally delivered** nCUR** alleviates clinical manifestations of colitis by efficiently scavenging ROS produced in the intestine. To investigate this therapeutic potential, C57BL/6 mice were given 3% DSS in their drinking water to induce intestinal colitis, as shown in **Figure 4A**. However, the mice were sensitive to successive DSS treatments, resulting in a high rate of mouse death (60%) and substantial body weight loss (**Figure 4B**-**C**). When we administered **nCUR** at a dose of 50 mg/kg (CUR equivalent dose) in the drinking water ad libitum, we surprisingly found that **nCUR** caused a substantial reduction in the mortality of mice with colitis. In addition, several typical symptoms of colitis, including diarrhea and rectal bleeding, were observed in the DSS-treated mice, whereas these symptoms were not observed in the **nCUR**-treated mice. Furthermore, we evaluated the severity of colitis on the basis of the DAI following 7 days of DSS treatment. Notably, compared with untreated mice with AOM/DSS-induced colitis, treatment with **nCUR** resulted in a significantly reduced average DAI; however, the therapeutic effect free CUR was negligible (**Figure 4D**).

Colon length is an important parameter for evaluating anti-inflammatory activity in the colitis model. As shown in **Figure 4E-F**, DSS treatment led to a marked shortening of the colon length. Interestingly, ad libitum administration of **nCUR** in the drinking water alleviated colitis-induced colonic shortening. For instance, the colon lengths in the mice receiving **nCUR** were 8.3 ± 1.1 cm, similar to the colon lengths in the healthy mice, whereas the average colon lengths in the DSS-induced colitis mice were only 6.2 ± 0.8 cm. Then, we performed histological analysis to examine the **nCUR** efficacy. Hematoxylin and eosin (H&E) staining revealed that the AOM/DSS treatment induced severe colitis in healthy mice. In colon sections, the occurrence of epithelial damage with abnormal crypts as well as the infiltration of macrophages and leukocyte into the colonic lamina propria were observed (**Figure 4G**). Interestingly, the damage and leukocyte infiltration induced by AOM/DSS was substantially reversed by the **nCUR** treatment, and the structural similarity of colonic crypts between **nCUR**-treated and healthy mice was observed. In this colitis model, disease severity is also correlated with high levels of proinflammatory cytokines. Thus, we analyzed several cytokines (proinflammatory mediators) to explore whether ad libitum drinking of **nCUR** exerts anti-inflammatory activity. Therefore, the total mRNA of colonic tissues was extracted from mice at day 20 and analyzed by quantitative RT-PCR. In AOM/DSS-induced colitis mice, oral administration of free CUR moderately suppressed these inflammatory cytokines (**Figure 4H**). Notably, the **nCUR** treatment substantially decreased inflammatory cytokines in colonic tissues, consistent with the observed *in vitro* anti-inflammatory effect. These results imply that **nCUR** ameliorates DSS-induced colitis in the murine CAC model.

### The nSN38 Nanotherapeutic Shows a Tumor-Targeting Ability in AOM/DSS-Induced CAC Mice

**Figure 5:**
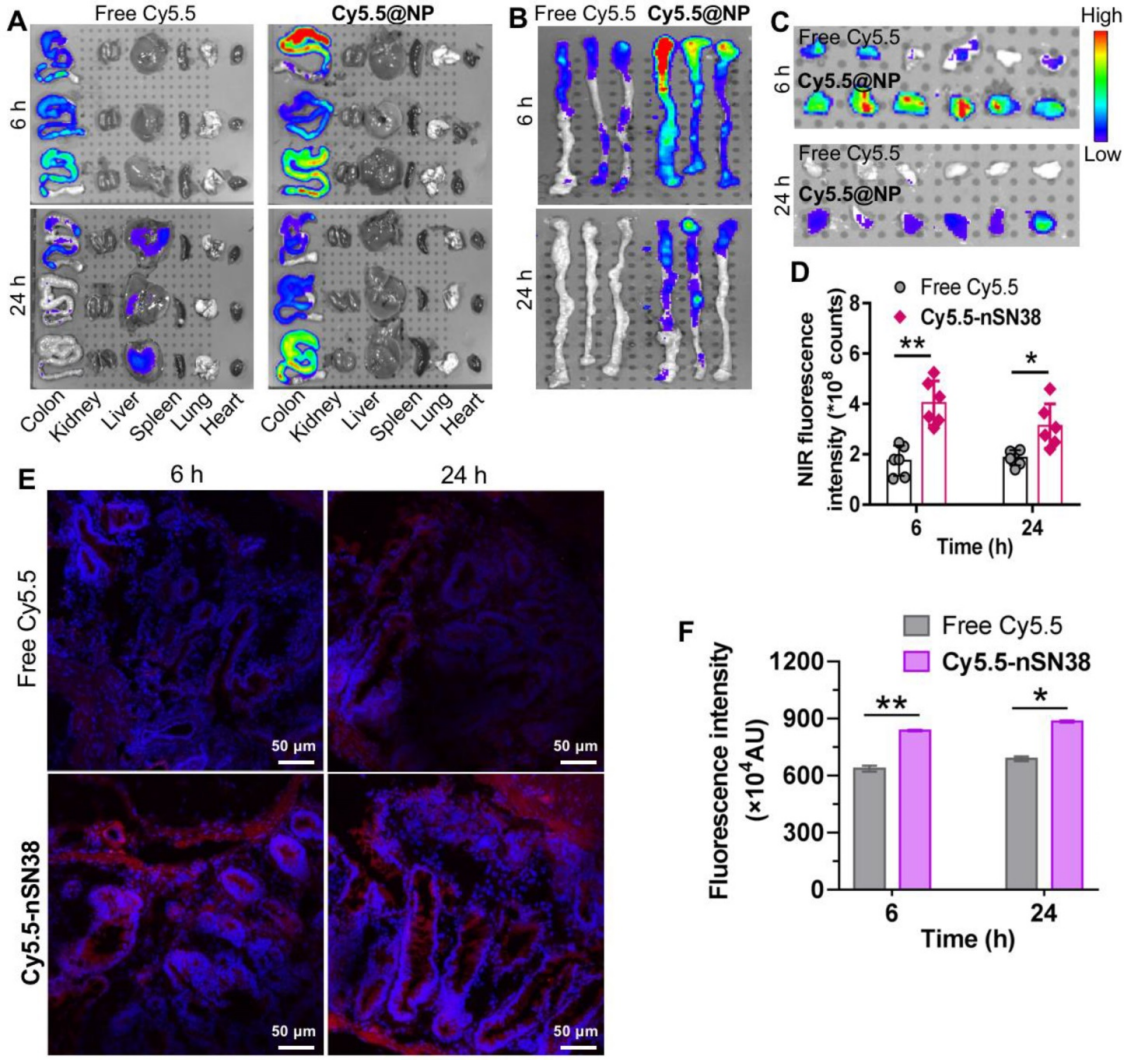
** Tumor-specific delivery of the nanotherapeutics in a CAC mouse model.** (**A**) Representative *ex vivo* fluorescence images for the evaluation of nanoparticle distribution in the major organs. C57BL/6 mice were intravenously injected with AOM followed by ad libitum administration of a 2% solution of DSS in drinking water for three cycles. This protocol allowed nearly all mice to develop tumors in the colonic tissues after 70 days. The mice with tumors were orally administered Cy5.5-labeled **nSN38** (termed **Cy5.5-nSN38**). A solution containing free Cy5.5 was also orally administered as a reference. (**B** and **C**) *Ex vivo* imaging of the whole colons (**B**) and tumors (**C**) excised from CAC mice at 6 and 24 h postadministration. (**D**) Quantification of fluorescence intensities of colorectal tumors. Confocal microscopy images (**E**) and fluorescence intensities (**F**) of colorectal tissue sections harvested at 6 and 24 h after oral administration. The red and blue signals indicate Cy5.5 and nuclei counterstained with DAPI, respectively. The data are presented as the mean ± SD. *p < 0.05 and **p <0.01, as determined by two-way ANOVA followed by Bonferroni's post hoc test.

Next, we investigated the tumor-specific accumulation ability of the nanotherapeutics in a CAC mouse model. AOM/DSS treatment generates colon tumors in healthy mice and closely recapitulates human CAC in terms of disease presentation and pathology. To establish the CAC model, C57BL/6 mice were intravenously injected with AOM followed by ad libitum drinking of 2% DSS in water for three cycles. This protocol allowed all mice to develop tumors in the colonic tissues after 70 days. Local delivery of cytotoxic drugs to tumors while sparing healthy tissues is a prerequisite to accomplish *in vivo* activity and reduce side effects. To track nanoparticle distribution, the NIR dye Cy5.5 was tethered to **nSN38** to yield Cy5.5-labeled **nSN38** (termed **Cy5.5-nSN38**). Free Cy5.5 formulated in polysorbate/ethanol (1:1, v/v) was also intragastrically administered to show the advantage of chitosan-based delivery. At 6 and 24 h postgavage, the major organs were excised, and *ex vivo* NIR fluorescence imaging was conducted (**Figure 5A**-**B**, and **Figure S10**). Strong signals derived from the Cy5.5 probe were solely observed in the colon regions and were not observed in the other organs in **Cy5.5-nSN38**-administered mice. In contrast, the Cy5.5 small-molecule dye showed negligible colon accumulation and faster clearance in the gastrointestinal tract. Furthermore, colorectal tumors were excised and then subjected to *ex vivo* imaging (**Figure 5C**). In contrast to free dye, **Cy5.5-nSN38** revealed specific localization within colonic tumors. Further quantitative analyses showed that oral administration of **Cy5.5-nSN38** led to 2.3- and 1.7-fold greater nanoparticle accumulation in tumors than free Cy5.5 in tumors at 6 and 24 h, respectively (**Figure 5D**). These results were further verified by fluorescence imaging of tumor sections using confocal microscopy (**Figure 5E**). Notably, at 6 and 24 h postadministration of the drug formulations, higher florescence signals were observed in colorectal tumors after treatment with** Cy5.5-nSN38** compared with free Cy5.5 (**Figure 5F**). Overall, these data provided compelling evidence that nanoparticles constructed from self-assembling chitosan-drug amphiphiles not only enabled the preferential localization in the inflamed regions but also accumulated in the tumor tissues after oral administration.

### The Synergy of nCUR/nSN38 in an AOM/DSS-Induced CAC Model

**Figure 6:**
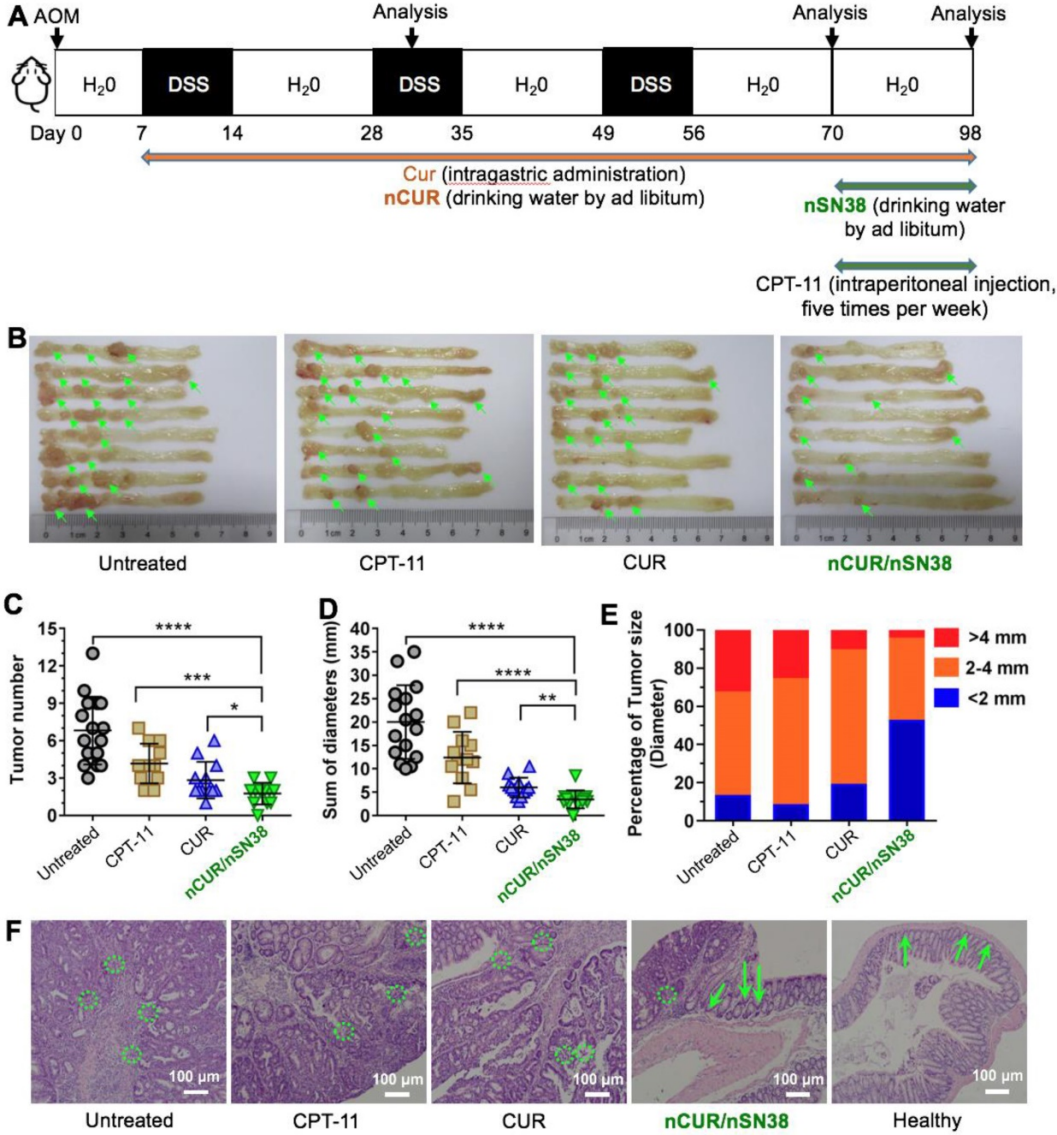
** The combination of nCUR/nSN38 nanotherapeutics prevents the tumorigenesis and growth of CAC *in vivo*.** (**A**) The establishment of an inflammation-driven colon cancer model in C57BL/6 mice and the therapeutic protocols of **nCUR/nSN38**. The mice received a single injection of AOM via the tail vein followed by supplementation with three cycles of 2% DSS. (**B**) Representative images of murine colons. (**C**-**E**) Tumor numbers per mouse (**C**), tumor diameters (**D**), and tumor size distribution (**E**) were measured in each group. (**F**) H&E staining of tumor morphology. The green arrows and dotted circles indicate crypts and leukocyte infiltration, respectively. The data are presented as the mean ± SD (n =12). *p < 0.05 and **p < 0.01, as determined by two-way ANOVA followed by Bonferroni's post hoc test.

Motivated by the results that **nCUR** ameliorates colitis in the murine CAC model and by the results showing long-term retention of **nCUR** in the gastrointestinal tract and the tumor tissue-specific targeting capability endowed by chitosan surface cloaking, we aimed to assess the therapeutic efficacy of using the combination of **nCUR/nSN38** in the CAC mouse model. Because **nCUR** was used as an anti-inflammatory agent, we initiated the therapy at day 7 following the AOM injection by ad libitum administration of **nCUR** (0.25 mg/mL in drinking water, equivalent to 50 mg/kg of CUR per day) (**Figure 6A**). The cytotoxic nanotherapeutic (i.e., **nSN38**, 0.1 mg/mL in water administered ad libitum, equivalent to 20 mg/kg of SN38 per day) was also orally administered in drinking water at day 70 and continued for 4 weeks. In this experimental setting, intragastric administration of CUR at a dose of 50 mg/kg per day and intraperitoneal injection of CPT-11 at a dose of 42 mg/kg for five times per week (equivalent to 20 mg/kg of SN38 per day) were included as references. At the end of the therapeutic study, the colons in each group were excised for assessing the efficacy. The colons in the mice that did not receive therapy showed the largest tumor volumes and numbers. Notably, the combinatorial therapy using **nCUR/nSN38** was more effective than **nCUR** or **nSN38** monotherapy, which resulted in markedly reduced tumor volumes and numbers in colonic tissues.

More specifically, the tumor growth inhibition (TGI) of the **nCUR/nSN38**-treated group was 94.9%, which is higher than **nCUR** or **nSN38** treatment with TGI of 60.3% and 73.3%, respectively (**Figure 6B**-**D** and **Figure S11** showing an independent experiment that proved the therapeutic synergy of the **nCUR/nSN38** treatment). Moreover, the CAC mice treated with **nCUR/nSN38** rarely had large tumors; for instance, tumors with a diameter exceeding 4 mm were only ~6% of the total tumors in these mice (**Figure 6E**). In contrast, ~35% of tumors were observed to exceed a diameter of 4 mm in the CAC mice. Notably, compared with soluble CPT-11 treatment, orally delivered **nCUR/nSN38** nanotherapeutics were also more effective in repressing tumor growth. These results indicate that reducing the severity of colitis by ad libitum drinking of **nCUR** in the initial stage of CAC is beneficial for the suppression of tumor growth.

Histopathological analysis further revealed that most of the tumors in the **CUR**-treated mice were adenomas with low-grade intraepithelial neoplasia and decreased leukocyte infiltration; however, only carcinoma tissues with seldom normal crypts and increased leukocyte infiltration were observed in the AOM/DSS-induced CAC mice (**Figure 6F** and **Figure S12**). In addition, a therapeutic synergy of using the **nCUR/nSN38** treatment was observed in the colons where the structure of crypts and leukocyte infiltration remained normal. Collectively, these results suggest the overall synergistic CAC treatment activity of the combination of **nCUR/nSN38** nanotherapeutics, which not only prevented colitis-associated tumorigenesis but also suppressed tumor growth in animals.

### The Combination of nCUR and nSN38 Inhibits the Progression of CAC by Inducing Cell Cycle Arrest and Apoptosis in Tumor Cells

**Figure 7:**
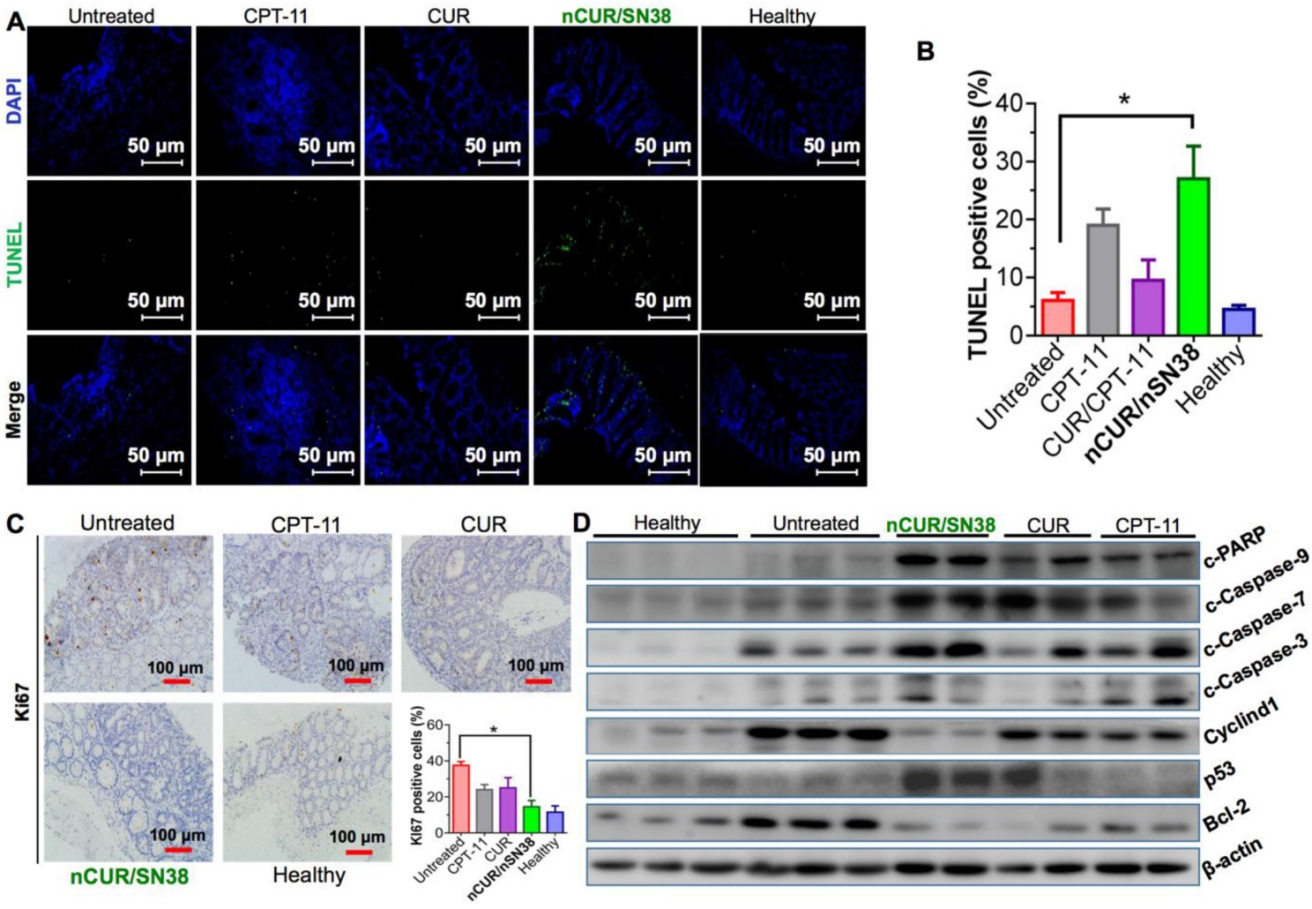
** The combination of nCUR/nSN38 inhibits the growth of CAC by inducing cell cycle arrest and apoptosis of tumor cells.** (**A**) TUNEL analysis of the excised tumors from each mouse group at the end of the therapeutic studies. (**B**) Quantification of apoptotic cells by counting TUNEL-positive cells. (**C**) Immunohistochemical staining of Ki-67 using paraffin-embedded tumor sections and quantification of positive Ki-67 staining. **(D)** The expression of indicated proteins isolated from the colonic epithelia of healthy mice and tumors of CAC mice after various treatments. The tumors excised from CAC mice without any therapeutic treatments were included as controls. *p < 0.05 and **p < 0.01, as determined by two-way ANOVA followed by Bonferroni's post hoc test.

Finally, we explored the mechanism of **nCUR/nSN38** for combinatorial efficacy in this preclinical CAC model. To examine the effect of **nCUR/nSN38** on cell apoptosis, a terminal deoxynucleotidyl transferase dUTP nick end labeling (TUNEL) assay was conducted. As shown in **Figure 7A**-**B**, **nCUR/nSN38** treatment led to a marked intratumoral apoptosis in the tumor tissues. These inhibitory effect was further validated by immunohistochemistry (IHC) staining of Ki-67 (**Figure 7C**). The results clearly showed that Ki-67 was mainly localized within the base of colonic crypts in healthy mice (**Figure 7C**). In contrast, in CAC mice, Ki-67 staining extended to most of the tumor area. Interestingly, compared with CPT-11 and CUR/CPT-11, the combination of the **nCUR** and **nSN38** nanotherapeutics significantly reduced the Ki-67-positive staining in tumor tissues. Moreover, western blotting was performed using excised tumor tissues to examine the molecular mechanism (**Figure 7D**). The AOM/DSS treatment resulted in the upregulation of cyclin D1 and cyclin D3 expression, which was significantly inhibited by **nCUR/nSN38** therapy. In addition, several proapoptotic proteins, including cleaved caspase 3 (c-caspase 3), cleaved caspase 7 (c-caspase 7), cleaved caspase 9 (c-caspase 9) and cleaved-PARP (c-PARP), were observed to be notably upregulated in the **nCUR/nSN38**-treated mouse tumors, indicating a much higher apoptotic rate in these tumors (**Figure 7D**). We additionally analyzed the expression of antiapoptotic proteins, such as Bcl-2, in isolated tumors. Consistently, a significant reduction in the expression level of Bcl-2 was observed in **nCUR/nSN38**-treated CAC tumor tissues. These results demonstrate that **nCUR/nSN38** suppresses the growth of AOM/DSS-induced CAC through the induction of cell cycle arrest and apoptosis in tumor cells.

### *In Vivo* Safety of Drug-Loaded Nanoparticles

Clinically, systemic administration of chemotherapy CPT-11 causes serious toxicity in patients with colorectal cancer. Enteritis, hematochezia, and diarrhea are the most common side effects, which impede long-term medications 8, 26. We anticipate that these orally deliverable nanoparticles have a high safety margin and could be used for the long-term treatment of CAC. To validate this assumption, we performed a toxicity study in healthy ICR mice (n = 5 mice in each group) with **nSN38** and **nCUR** orally administered. The mice treated with CPT-11 and saline were used as controls. As shown in **Figure S13**, only the mice that received intraperitoneal injections of CPT-11 exhibited a substantial drop of body weights as well as an escalated level of DAI. Impressively, in the mice orally receiving **nSN38** and **nCUR**, no difference in body weight or DAI was observed compared with saline-treated group. Furthermore, we performed histological analyses of digestive organs excised from the mice. CPT-11 elicited colon damages as measured by H&E staining (**Figure S14**). Notably, no signs of necrosis and cell death in organs of **nSN38**- and **nCUR**-treated mice were observed. Therefore, these toxicity assessments supported the *in vivo* safety of nanotherapeutics.

## Discussion

IBDs are inflammatory disorders of the intestinal tract and are the highest risk factors for colorectal cancer. The pathogenesis of IBD remains poorly explored 3, 32, 33. Elevated levels of ROS and an imbalance of antioxidants in the intestinal mucosa of IBD patients have been implicated in the development and progression of IBDs 34. In preclinical mouse models, antioxidant agents and free radical scavengers have been shown to ameliorate colitis 35, 36. Currently, several regimens, including salicylic acid, glucocorticoids and immunosuppressive therapies, are used for treating IBD in the clinic 37, 38. Unfortunately, these therapies have shown limited outcomes in terms of preventing colitis, presumably due to low bioavailability, poor selectivity and retention at inflammatory sites, and severe side effects caused by long-term medications. In addition, intravenous injections are generally required for these therapeutics, resulting in severe side effects and decreased patient compliance. Considering these characteristics, including slow progression and recurrence, oral administration of therapeutics could favor the long-term management of chronic IBDs. In line with this, a collection of nanotherapeutics have been developed for oral delivery of therapeutics to diseased gastrointestinal sites because of the convenience and compliance by patients 39-42.

CUR is extracted from *Curcuma longa* and has numerous pharmacological activities. Owing to its anti-inflammatory activity, the potential of CUR for the prevention and treatment of colitis warrants investigation. However, CUR has poor water solubility and absorption in the gastrointestinal tract after oral administration. Unlike small-molecule drugs, orally administered nanoparticles generally exhibit specific accumulation in inflamed tissues *via* the enhanced permeability and retention (EPR) effect 43. Therefore, we hypothesized that by using bioadhesive materials as carriers or for surface cloaking, intestinal retention and absorption of therapeutic drugs can be improved. Previous studies have suggested that low-molecular-weight chitosans contribute to enhanced oral absorption and are capable of transporting attached drug payloads across intestinal epithelial cells 19, 20. More interestingly, low-molecular-weight chitosans generally have negligible cytotoxicity according to *in vitro* cell-based assays. These characteristics make them ideal scaffold materials for drug conjugation and oral delivery. In this study, we coupled the anti-inflammatory agent CUR to mucoadhesive chitosan to enhance accumulation in inflamed lesions. The hydrophobicity of CUR provided the overall chitosan-CUR amphiphile with the ability to self-assemble into stable core-shell nanostructures in water. Therefore, **nCUR** is well suited for oral administration applications for *in vivo* therapeutic studies.

Previous studies have demonstrated that inhibiting the expression of inflammatory factors secreted by macrophages is crucial for the prevention and treatment of CAC 44, 45. In the cell-based assays, we found that treatment with **nCUR** reduced inflammatory cytokines (e.g., IL-1β, IL-6, IFN-γ and iNOS) and ROS in Raw264.7 cells (**Figure 2**). More interestingly, the addition of **nCUR** reversed the increase in inflammatory cytokines induced by SN38 treatment *in vitro* (**Figure 2**). Furthermore, in mice with colitis, treatment with **nCUR** alleviated colonic inflammation, as indicated by improved mouse survival, normalized colon length and reduced inflammatory cytokines in colon tissues. Overall, these results indicate that orally administered **nCUR** has the ability to ameliorate DSS-induced intestinal inflammation, potentiating the combinatorial synergy with the subsequent treatment with **nSN38** to reduce the incidence of developing CAC.

One of the consequences of chronic IBDs is the promotion of tumorigenesis, and patients with IBD have a higher risk of developing CAC. Long-term and repeated intravenous injections of chemotherapeutic drugs can lead to potential systemic toxicity. In addition, IBDs typically have characteristics of recurrence and persistence. A considerable number of patients suffer from unsatisfactory treatments and eventually develop CAC after more than 10 years 46, 47. Therapies, including irinotecan, oxaliplatin, 5-fluorouracil/leucovorin, and bevacizumab, have been used in single and combined administrations as anticancer drugs in the late stage of CAC 6, 7, 48. Therefore, there is a considerable incentive for the development of orally bioavailable nanotherapeutics that are capable of delivering therapeutic agents precisely and exclusively to inflamed regions for long-term treatment. This type of nanosystem could ameliorate the side effects of existing therapeutics; however, the development of these types of systems poses challenges. Therefore, oral administration is a favorable route for the long-term treatment of gastrointestinal diseases linked to intestinal inflammation and cancers.

Exploiting carboxyl-derived chitosan, a potent DNA topoisomerase I inhibitor SN38 was integrated for localized treatment of CAC. Similar to CUR, SN38 is not soluble in aqueous media. Poor absorption in the gastrointestinal tract hampers its oral administration. The water-soluble prodrug CPT-11 has been developed as a standard-of-care therapy for colorectal cancer, but the clinical efficacy is largely limited due to low enzymatic conversion to the therapeutically active SN38 molecule (generally <8%) 48. In addition, systemic administration of CPT-11 *via* the intravenous route is often accompanied by adverse reactions, such as enteritis, hematochezia, diarrhea, and vomiting, which impair long-term treatment of patients with CAC. Notably, SN38 is highly potent in inducing cancer cell apoptosis and has 100- to 1000-fold greater cytotoxicity than CPT-11 according to cell-based assays 49. Therefore, directly harnessing SN38 could enhance the therapeutic index of this agent for cancer therapy.

In our previous work, we showed that the potency of the SN38 agent can be significantly augmented through rational polylactide engineering and sequential nanoparticle assembly 13, 50. In the present study, the active SN38 agent was covalently attached to mucoadhesive chitosan without a tedious synthetic procedure. The resulting chitosan-SN38 amphiphile self-assembled into an orally absorbable nanotherapeutic (i.e., **nSN38**) for the treatment of AOM/DSS-induced tumor-bearing mice. In addition to accumulation in inflamed intestines, the chitosan scaffold was found to preferentially localize in tumor tissues in CAC mice (**Figure 5**). Notably, we validated that **nSN38** had relatively longer retention in colons than free molecules, presumably due to the mucoadhesive property provided by chitosan. After being taken up by cancer cells, **nSN38** releases chemically unmodified drugs because of the self-hydrolyzable ester linkage. Notably, compared with individual drug treatments, the synergy of the combination of **nCUR/nSN38** was confirmed, as indicated by the reduced average tumor numbers and sizes compared (**Figure 6**). Therefore, ameliorating colitis by oral administration of **nCUR** in drinking water in the initial stage of CAC is beneficial for the suppression of tumor growth.

## Conclusion

Oral administration is the most convenient and cost-effective approach to deliver drugs with low bioavailability to diseased intestinal tissues for anti-inflammatory and anticancer therapy. In this study, we developed orally deliverable nanotherapeutics for the synergistic treatment of CAC in preclinical mouse models. Water-insoluble CUR and SN38, which served as anti-inflammatory and anticancer agents, respectively, were rationally engineered in a bioadhesive chitosan scaffold to assemble colloidal-stable nanoparticles. The synergy of **nCUR** and **nSN38** was further confirmed* in vitro* and *in vivo*, and these drugs were shown to provide a reliable oral administration strategy for the simultaneous treatment of IBDs and related colorectal cancer, thus warranting further investigation. On the basis of chitosan platforms, other therapeutic agents that are not capable of oral administration could be reasonably re-engineered for oral administration 51. We expect that this oral nanotherapeutic approach could make a substantial contribution to the management of numerous gastrointestinal diseases, including IBDs, gastrointestinal cancers, and various viral infections.

## Supplementary Material

Supplementary figures and tables.
