# Supplementary material for: Orally Deliverable Nanotherapeutics for the Synergistic Treatment of Colitis-Associated Colorectal Cancer
Source: Theranostics. 2019 Oct 12;9(24):7458–73. doi: 10.7150/thno.38081 (PMC6831307; doi:10.7150/thno.38081)
Supplement: Supplementary file 1 — Supplementary figures and tables. [file thnov09p7458s1.pdf]

Supplementary Material

Orally Deliverable Nanotherapeutics for the Synergistic Treatment of Colitis-Associated Colorectal Cancer

Weidong Han,[a,*] Binbin Xie,[a] Yiran Li,[a] Linlin Shi,[a] Jianqin Wan,[b] Xiaona Chen,[b] Hangxiang Wang[b,*]

[a]Department of Medical Oncology; Sir Run Run Shaw Hospital; School of Medicine, Zhejiang University, Hangzhou, 310016, PR China.

[b]The First Affiliated Hospital; Key Laboratory of Combined Multi-Organ Transplantation, Ministry of Public Health; School of Medicine, Zhejiang University, Hangzhou, 310003, PR China.

Corresponding Authors: Hangxiang Wang, The First Affiliated Hospital, School of Medicine, Zhejiang University; 79, Qingchun Road, Hangzhou 310003, China. Phone: +86-571-88208173; Fax: +86-571-88208173; E-mail: wanghx@zju.edu.cn

Corresponding Authors: Sir Run Run Shaw Hospital; School of Medicine, Zhejiang University, Hangzhou, 310016, PR China. Weidong Han, hanwd@zju.edu.cn

Materials and Methods

Synthesis of *N*-carboxyethylchitosan

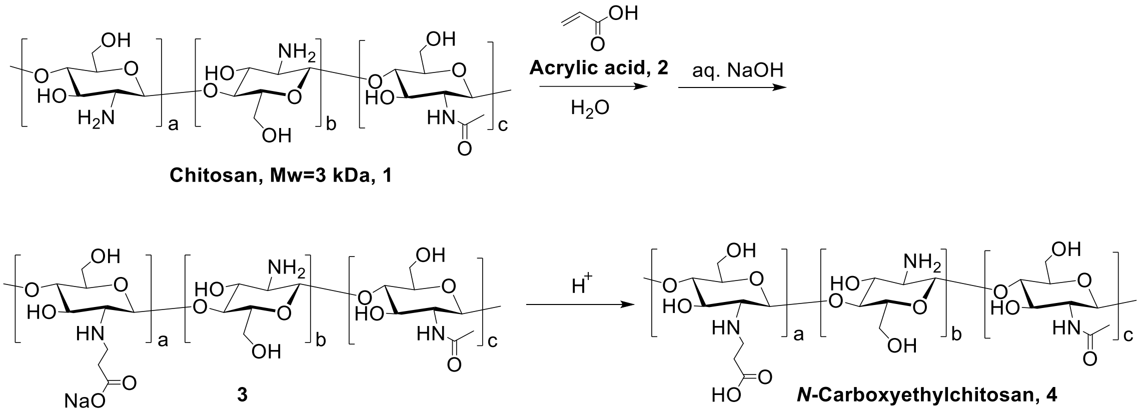

Synthetic scheme S1.

N-Carboxyethylchitosan was synthesized using the Michael reaction according to the previously reported protocol. Low molecular weight chitosan (Jinan Haidebei Marine Bioengineering Co. Ltd., China, average Mw=5,000 Da, 16.3 g, corresponding to 90 mmol NH_2) was dissolved in water (500 mL) containing acrylic acid (25 mL, 360 mmol, 4.0 equiv./NH_2). The reaction mixture was refluxed. After three days, aqueous NaOH (1 M) was added to the solution to adjust the pH to 10–12 to convert the carboxylic acid to its sodium salt. The mixture was dialyzed against phosphate buffer saline (PBS, pH 8.0) using a dialysis tube (Spectrum, molecular weight cutoff of 14 kD) to remove unreacted acrylic acid for two days and then dialyzed against deionized water for one day. Sodium cation was removed by cation exchange resin (Diaion WK-40, Mitsubishi Chemical Corporation). Finally, the solution was lyophilized to obtain *N*-carboxyethylchitosan, **4**.

Synthesis and self-assembly of the chitosan-curcumin conjugate to form nCUR

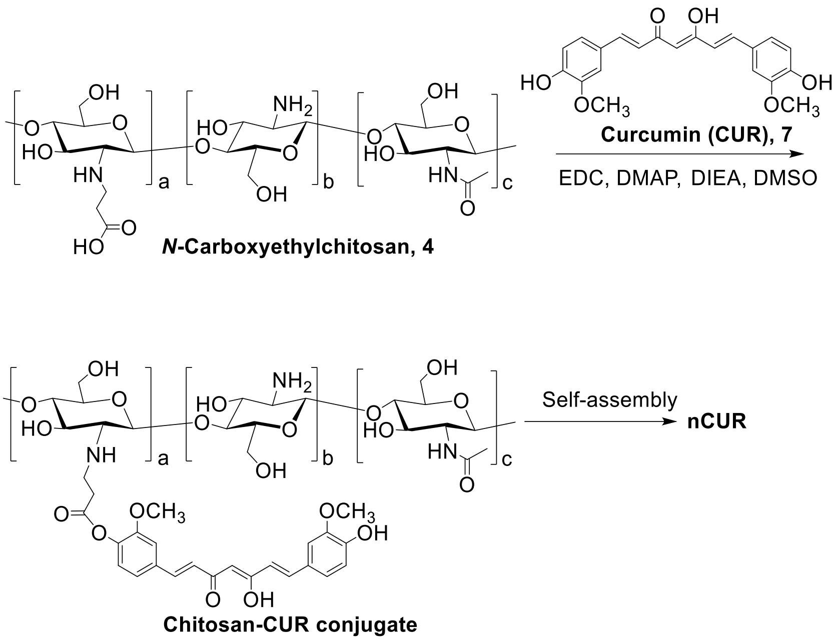

Synthetic scheme S2.

To a solution of *N*-carboxyethylchitosan (168 mg) in dry dimethyl sulfoxide (DMSO, 10 mL) was added curcumin (CUR, 300 mg, 0.81 mmol), 1-ethyl-3-(3-dimethylaminopropyl)carbodiimide (EDC, 129 mg, 0.81 mmol), 4-dimethylaminopyridine (DMAP, 101 mg, 0.81 mmol) and *N,N*-diisopropylethylamine (DIEA, 133 mg, 1.02 mmol). This reaction allowed formation of the ester linkage between chitosan and CUR. The reaction mixture was stirred for 1 day at 50°C, and then dialyzed for 3 days in water/methanol mixture (1:4 v/v) using a dialysis tube (Spectrum, molecular weight cutoff of 14 kD) to remove excessive CUR. Finally, the solution was further dialyzed for 1 day in deionized water and lyophilized to afford chitosan-curcumin conjugate-assembled nanoparticles (**nCUR**).

Synthesis and self-assembly of the chitosan-SN38 conjugate to form nSN38

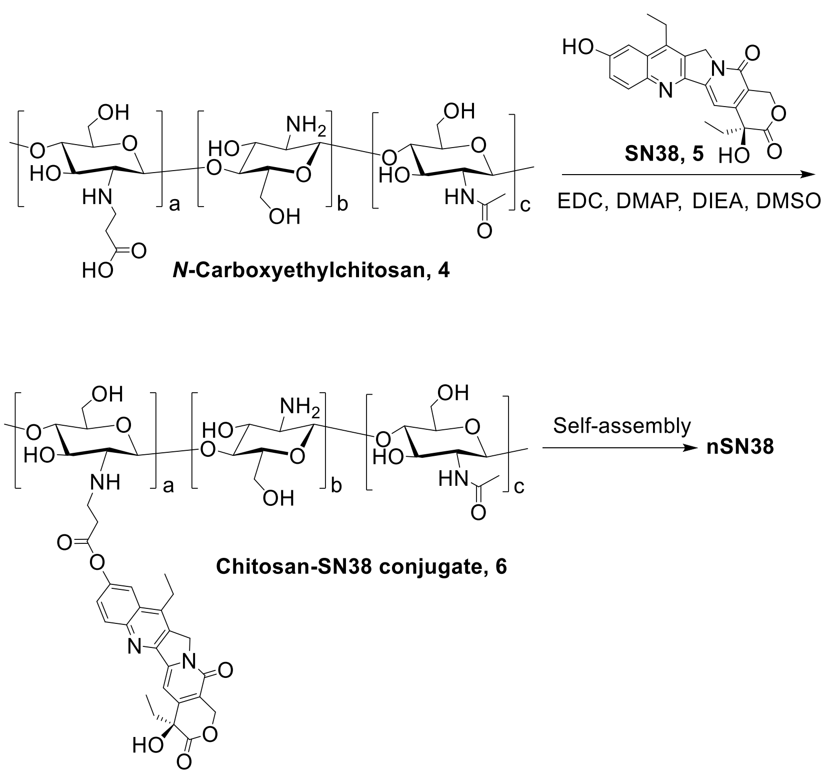

Synthetic scheme S3.

To a solution of *N*-carboxyethylchitosan (156 mg) in dry DMSO (10 mL) was added 7-ethyl-10-hydroxycamptothecin (SN38, 300 mg, 0.76 mmol), EDC (120 mg, 0.76 mmol), DMAP (93 mg, 0.76 mmol) and DIEA (124 mg, 0.95 mmol). This reaction allowed formation of the ester linkage between chitosan and SN38. The reaction mixture was stirred for 1 day at 50°C, and then dialyzed for 3 days in water/methanol mixture (1:4 v/v) using a dialysis tube (Spectrum, molecular weight cutoff of 14 kD) to remove excessive SN38. Finally, the solution was further dialyzed for 1 day in deionized water and lyophilized to afford chitosan-SN38 conjugate-assembled nanoparticles (**nSN38**).

Morphology study using transmission electron microscopy (TEM) analysis

TEM images were obtained using TECNAL 10 (Philips) at an acceleration voltage of 80 kV. The sample solution of SN38 or curcumin-loaded nanoparticles at a concentration of 0.5 mg/mL (drug equivalent) was placed onto a 300-mesh copper grid coated with carbon. Approximately 2 min after deposition, the surface

water was removed with filter paper and then air dried. Positive staining was performed using a 2 wt% aqueous uranyl acetate solution.

Dynamic light scattering (DLS) analysis

The concentration of drugs in nanoparticle solutions was adjusted to approximately 0.1 mg/mL. Hydrodynamic diameters (D_H), distribution and zeta potentials were determined by dynamic light scattering (Malvern Nano-ZS90 instrument, Malvern, UK).

In vitro drug release kinetics study

Simulated gastric fluid (SGF) and simulated intestinal fluid (SIF) were prepared according to the previously reported recipes [1]. SGF: 0.2 g NaCl and 7.0 mL HCl (1.0 mol/L) were added to deionized water (1.0 L, pH 1.2). SIF: 6.8 g KH_2PO_4 and 0.896 g NaOH were added to deionized water (1.0 L, pH 6.8).

Drug release profiles were examined with the dialysis method to quantify the drug release behavior. The solutions containing with **nSN38** or **nCUR** with a 0.1 mg/mL drug equivalent concentration were loaded into dialysis bags (Spectrum, molecular weight cutoff of 7 kD). The solutions were dialyzed against PBS (pH 7.4, 0.2% Tween 80), SGF (pH 1.2, 0.2% Tween 80), or SIF (pH 6.8, 0.2% Tween 80). The dialysis bags were continuously and gently shaken in an orbital shaking water bath at 37°C, and the release media were collected and supplemented with fresh media at predetermined time intervals. The amounts of released SN38 or CUR were analyzed using a UV-vis spectrometer (Shimadzu, UV-2700).

Stability of SN38- or CUR-loaded nanoparticles in gastrointestinal environment

The solutions containing with **nSN38** or **nCUR** were diluted 10-fold with SGF or SIF to a final concentration at 0.1 mg/mL (SN38- or CUR-equivalent concentration), followed by incubation at 37°C for 24 h. The particle sizes and zeta potentials were examined by DLS measurement at predetermined time intervals.

Mice colon tissue fixation and histology processing

After mice were euthanized, the entire colon was obtained, and the fecal contents from the large intestine was removed. Subsequently, the distance between the ileocecal junction and the anus was measured. After cutting open the intestinal tract longitudinally, the number and size of the tumors were quantified and recorded. Distal colons were fixed in 4% paraformaldehyde for 24 h, and used to make the colon slides for hematoxylin-eosin (H&E), immunohistochemistry (IHC) and the terminal deoxyribonucleotidyl transferase (TDT)-mediated dUTP-digoxigenin nick end labeling (TUNEL) staining. The mid-colons were utilized for western blot and RNA extraction.

RNA extraction, reverse transcription and real-time PCR

Total RNA was extracted using TRIzol reagent (Takara, Kyoto, Japan) followed by measuring the RNA concentration using a NanoDrop 2000c spectrophotometer (Thermo Scientific, USA). cDNA from RAW264.7 cells, BMDMs and murine colon tissues was synthesized using an PrimeScript™ RT reagent Kit (Takara, Kyoto, Japan). Quantitative real-time PCR (qPCR) was performed using TB Green Premix Ex Tak (Takara, Kyoto, Japan). Primer sequences are listed in Table S1. The target gene expression was analyzed using the 2-ΔΔCq method [2, 3] and normalized to the reference gene GAPDH.

Immunohistochemistry (IHC)

After deparaffinization, the mouse colon and tumor sections were incubated with 10 mM sodium citrate buffer (pH 6.0) for antigen retrieval, Endogenous peroxidase was conducted with 3% hydrogen peroxide. The slides were incubated overnight at 4°C with the following primary antibodies: Ki67 (Abcam, 1:100, ab16667) and c-PARP (Abcam, 1:100, ab32064). The secondary antibody conjugated with horseradish peroxidase (HRP) -labeled goat anti-rabbit IgG antibody (ZSGB-BIO, Beijing, China) was used for incubation with the slides at room temperature for 1 h. The slides were also counterstained with hematoxylin.

Western blot

Briefly, the extracted protein of cell and mouse colon samples were prepared and separated on SDS-PAGE gels. After the electrophoresis, the separated proteins were transferred to PVDF membranes (Millipore, Billerica, MA, USA) and blocked with 5% non-fat milk dissolved in Tris-buffered saline with Tween (TBST) for 1 h at room temperature. After washing in TBST, the membrane was incubated with primary and secondary antibodies (Jackson ImmunoResearch, PA, USA). Primary antibody information was shown in Table S2. Signals were visualized with enhanced chemiluminescence (Fude Biological Technology, Hangzhou, China).

TUNEL staining

TUNEL assay was performed according to instrument of the In situ cell death detection kit (Roche, Germany). Tissue sections were dewaxed and rehydrated, Proteinase K working solution was added and incubated for 30 min at 37°C. After being rinsed twice with PBS, the slides were incubated with TUNEL reaction mixture for 60 min at 37°C. Cell nuclei were then stained with 4',6-diamidino-2-phenylindole (DAPI) and fluorescence imaging was visualized under a Leica DMIL LED microscope (Leica Microsystems). Five random selected fields from each sample were counted in a single blinded manner.

5-ethynyl-2-deoxyuridine (EdU) assay

EdU Cell Proliferation Kit with Alexa Fluro 488 (C0081S, Beyotime Biotechnology) was used to detect and quantify cell proliferation in live cells. HCT116 and DLD1 cells (1×10^6 cells/well) were seeded in 6-well plates for overnight. Thereafter, the cells were exposed to the same concentrations (20 nM) of drugs (CPT-11, SN38, and **nSN38**) for 48 h. After drug treatment, the cells were incubated with EdU (10 μM) for 2 hours at 37°C. Then, the cells were trypsinized, extracted and washed for subsequent fixation and permeation. After staining with the click reaction liquid for 30 minutes in the dark at room temperature, the samples were analyzed using flow cytometry (BD LSRFortessa, USA).

Apoptosis assay

Annexin V- FITC/PI Apoptosis Detection Kit (MA0220, Meilun Biotechnology Co., Ltd.) was used to assess the apoptotic rate of cancer cells. Briefly, 1×10^6 cells per well were seeded in 6-well plates overnight and exposed to the same concentrations (20 nM) of drugs (CPT-11, SN38, and **nSN38**) for 48 h. Thereafter, the cells were collected by trypsinization, washed twice with cold PBS and resuspended in 500 μL 1×binding buffer. Next, the cells were mixed with 5 μL FITC Annexin V and 5 μL propidium iodide and incubated for 15 minutes in the dark at room temperature. After incubation, the levels of apoptosis were analyzed with flow cytometry (BD LSRFortessa, USA). Untreated cells suspended in PBS were used as the control.

Evaluation of the drug toxicity in healthy ICR mice

The *in vivo* toxicity of **nSN38** and **nCUR** was evaluated in healthy ICR mice (6 weeks old). The mice were intraperitoneally injected with CPT-11(42 mg/kg, five times for every week). **nSN38** (20 mg/kg per day, 0.1 mg/mL) and **nCUR** (50 mg/kg per day, 0.2 mg/mL) were administered ad libitum in water. We recorded the body weight every two days. Disease activity index (DAI) was assessed on day 14. After two weeks, the organs of stomach, small intestine and colon were excised and subjected to histological analysis (i.e., H&E staining) to examine the damage caused by the drugs.

Table S1. Real-time PCR primers

Genes	Real time PCR primers sequence
IL-1β	F: 5'- GTGGCTGTGGAGAAGCTGTG -3'
	R: 5'- GAAGGTCCACGGGAAAGACAC -3'
IL-6	F: 5'- CTCTGCAAGAGACTTCCATCCAGT-3'
	R: 5'- GAAGTAGGGAAGGCCGTGG-3'
IFN-β	F: 5'- CAGCTCCAAGAAAGGACGAAC-3'
	R: 5'- GGCAGTGTAACTCTTCTGCAT-3'
TNF-α	F: 5'- AGGGTCTGGGCCATAGAACT-3'
	R: 5'- CCACCACGCTCTTCTGTCTAC-3'
iNOS	F: 5'- GAGCTTCTACCTCAAGCTATC-3'
	R: 5'- CCTGATGTTGCCA-TTGTTGGT-3'
MCP1	F: 5'- TTAAAAACCTGGATCGGAACCAA-3'
	R: 5'- GCATTAGCTTCAGATTTACGGGT-3'
GAPDH	F: 5'- TTGATGGCAACAATCTCCAC-3'
	R: 5'- CGTCCCGTAGACAAAATGGT-3'

Table S2. Antibodies used in this study

Antibody	Supplier	Cat. No.	Host	Dilution
c- PARP	Cell Signaling Technology	9548	Rabbit	1:1000
c-Caspase-9	Cell Signaling Technology	9509	Rabbit	1:1000
c-Caspase-7	Cell Signaling Technology	9491	Rabbit	1:1000
c-Caspase-3	Cell Signaling Technology	9664	Rabbit	1:1000
Cyclind1	Cell Signaling Technology	2926	Mouse	1:1000
P53	Cell Signaling Technology	9282	Rabbit	1:1000
Bcl-2	Cell Signaling Technology	3498	Rabbit	1:1000
β-actin	Cell Signaling Technology	4967	Rabbit	1:1000

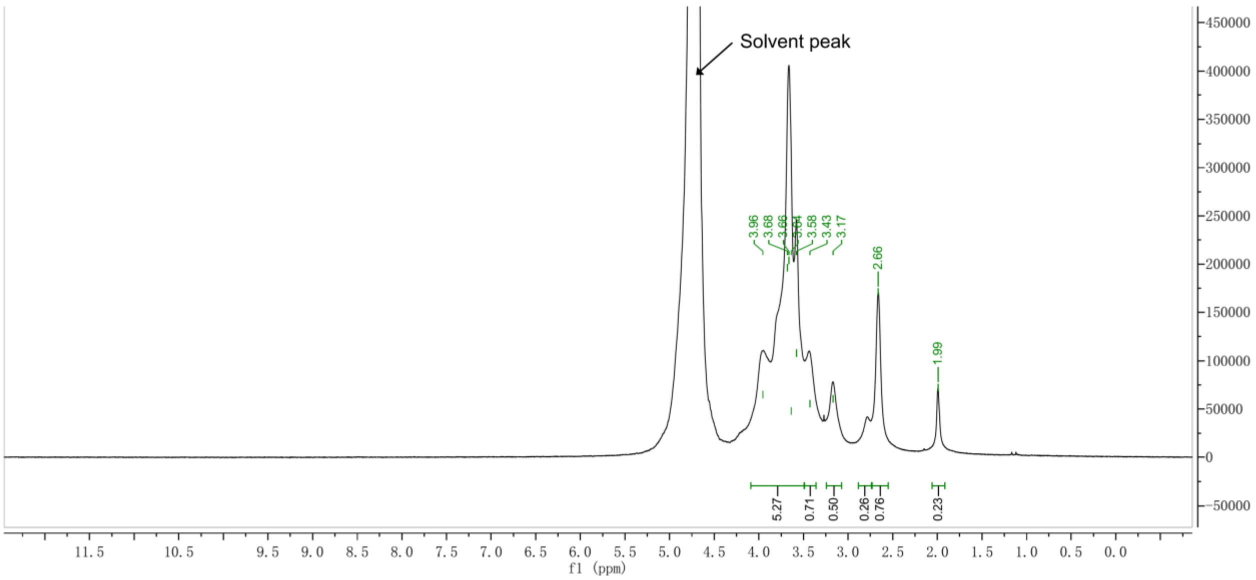

Figure S1. ^{1}H NMR spectrum of *N*-carboxyethylchitosan in D_2O.

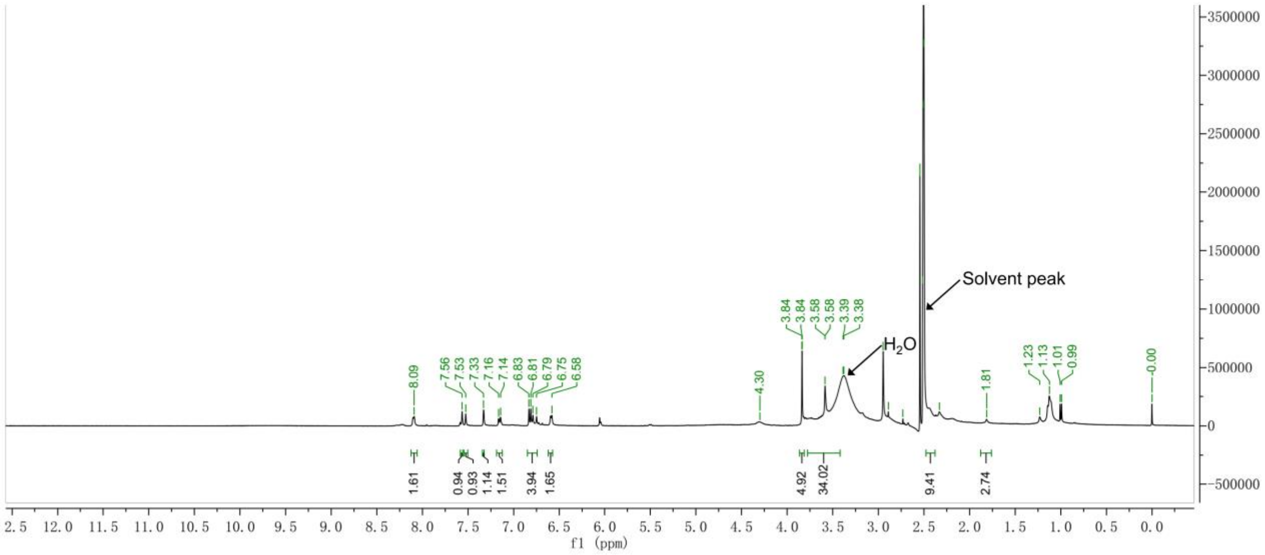

Figure S2. ^{1}H NMR spectrum of the chitosan-curcumin conjugate in DMSO-d_6.

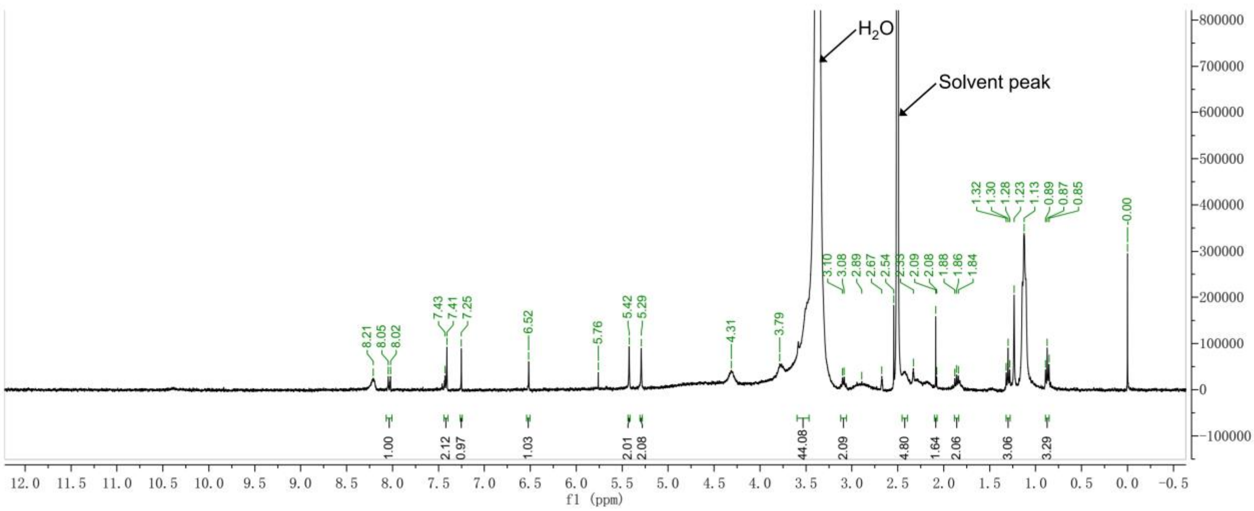

Figure S3. ^{1}H NMR spectrum of the chitosan-SN38 conjugate in DMSO-d_6.

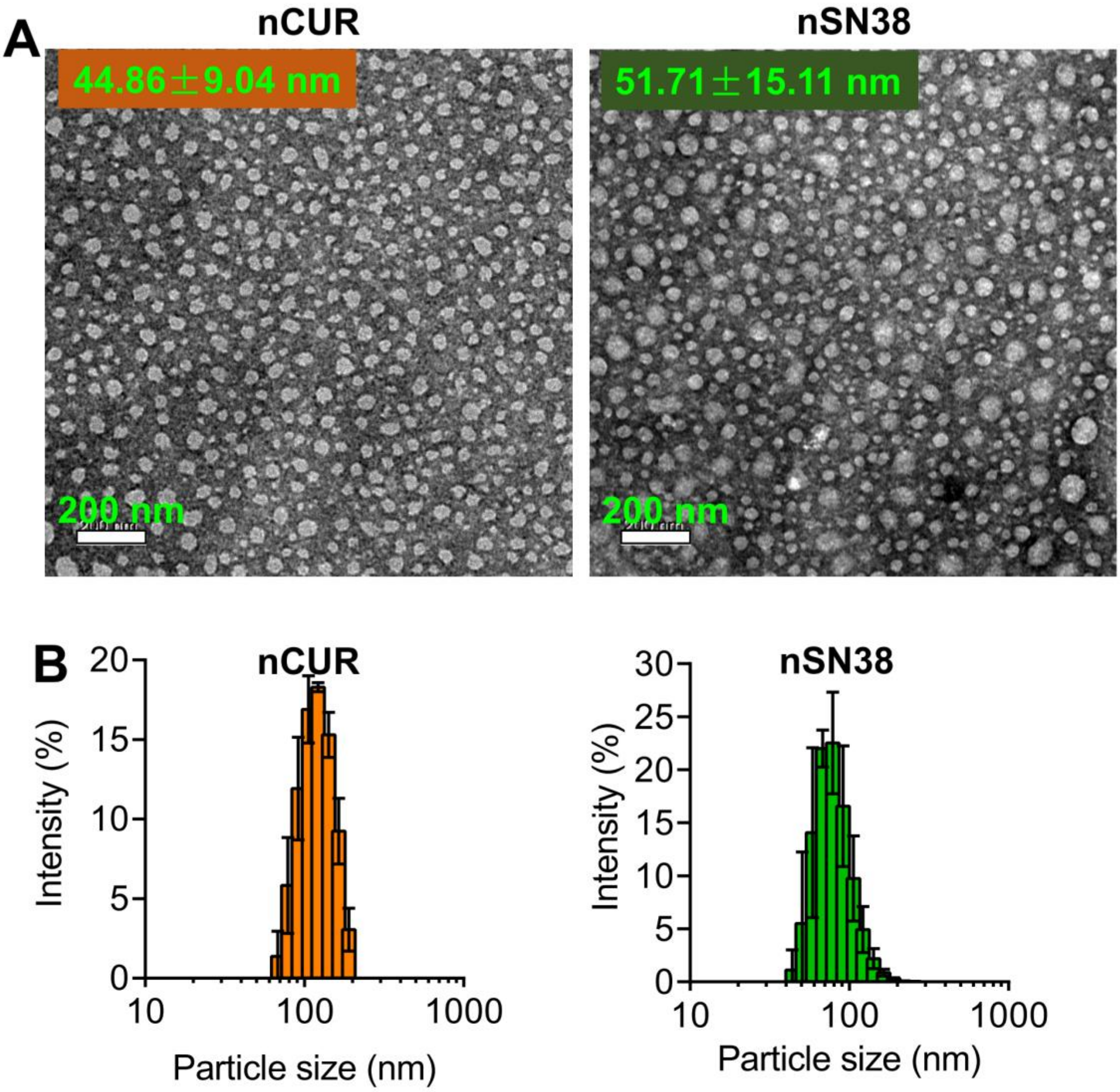

C

	D_H (nm)	PDI	Zeta potential (mV)
**nSN38**	118.1±87.7	0.317±0.004	-15.2±0.9
**nCUR**	81.2±41.3	0.130±0.001	-27.4±0.3

Figure S4. Characterization of self-assembled chitosan-drug conjugate nanoparticles. (**A**) Transmission electron microscopy (TEM) images of **nCUR** and **nSN38**. (**B**) Size distribution of nanotherapeutics as determined by dynamic light scattering (DLS). (**C**) Hydrodynamic diameters (D_H) and zeta potentials of drug-loaded nanotherapeutics in deionized water. The NP suspensions containing 0.1 mg/mL of CUR- or SN38-equivalent concentration were used. The data are presented as the mean ± SD (n = 3).

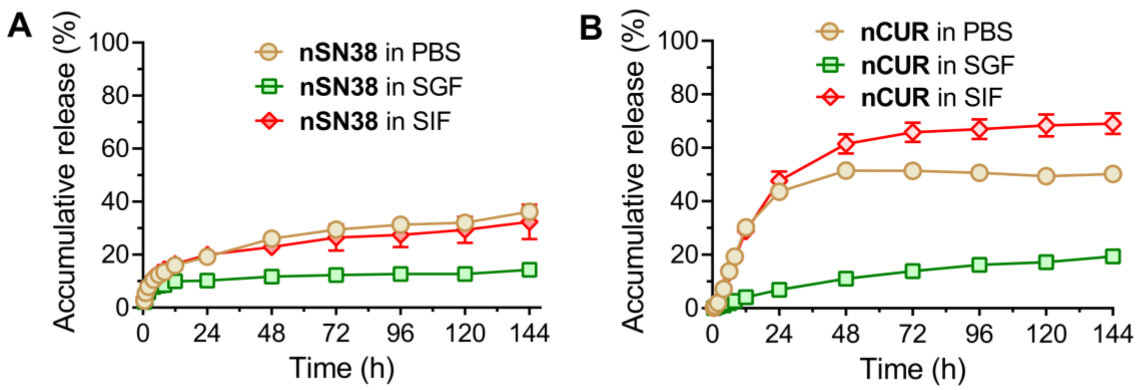

Figure S5. *In vitro* drug release profiles of SN38 (**A**) and CUR (**B**) from **nSN38** and **nCUR**, respectively. The NP suspensions (at a 0.1 mg/mL of drug-equivalent concentration) were dialyzed against PBS (pH 7.4), SGF (pH 1.2), or SIF (pH 6.8) over a 144-h period at 37°C. All dialyzing solutions contained 0.2% Tween 80. The data are presented as the mean ± SD (n = 3).

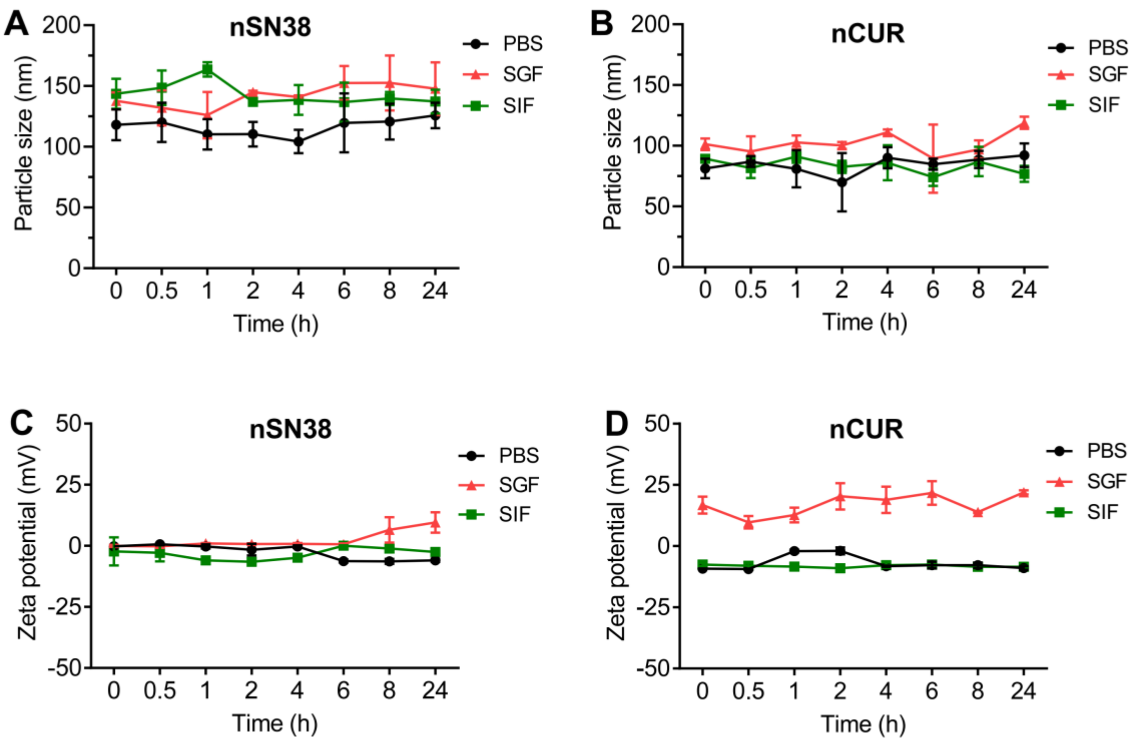

Figure S6. Stability of nanotherapeutics in different media as determined by DLS measurement. Changes of D_H (**A** and **B**) and zeta potentials (**C** and **D**) in PBS (pH 7.4), SGF (pH 1.2), or SIF (pH 6.8).

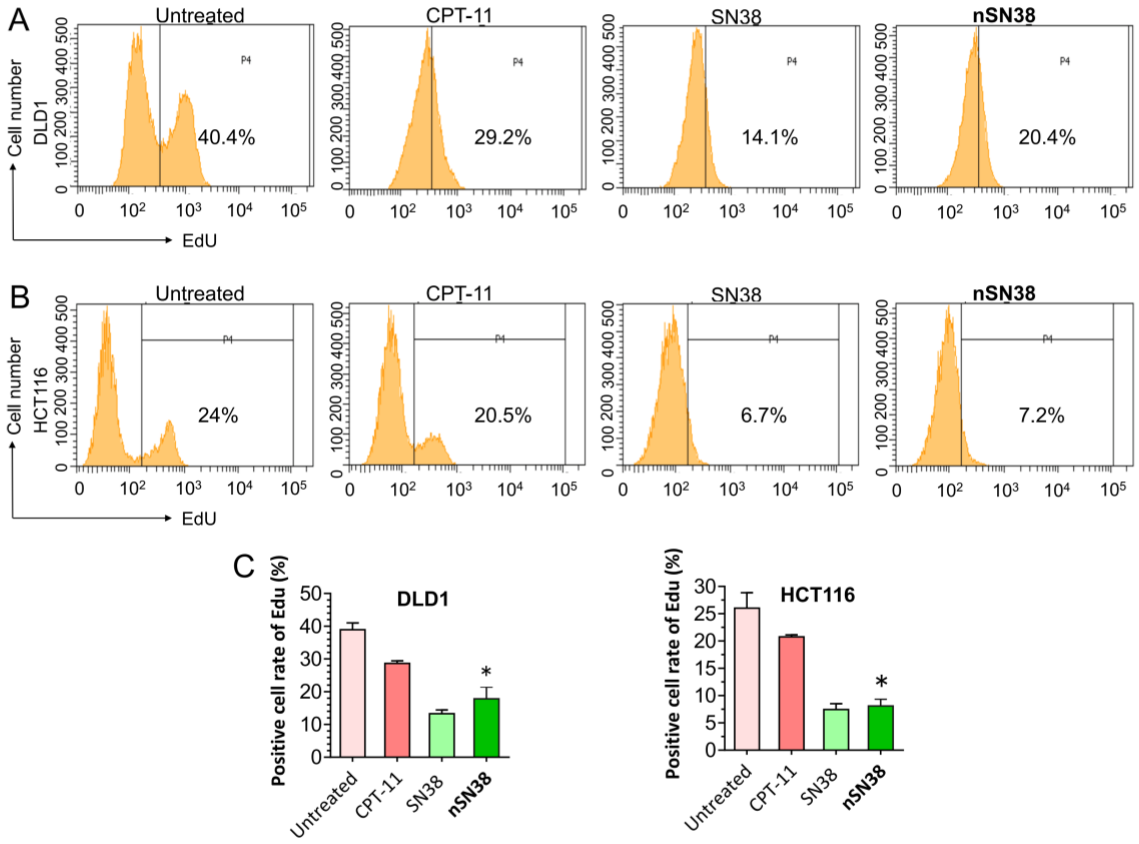

Figure S7. The *in vitro* cytotoxicity against colon cancer cell lines. Analysis of drug effect on the proliferation of DLD1 (**A**) and HCT116 (B) cells using an EdU Cell Proliferation Kit. (**C**) The percentage of EdU positive cells in each group is shown in the histogram. The cells were treated with the same concentrations (10 nM) of CPT-11, SN38, and **nSN38** for 48 h. Untreated cells were included as controls. The data are presented as the mean ± SD (n=3); *$p < 0.05$ as compared to untreated cells.

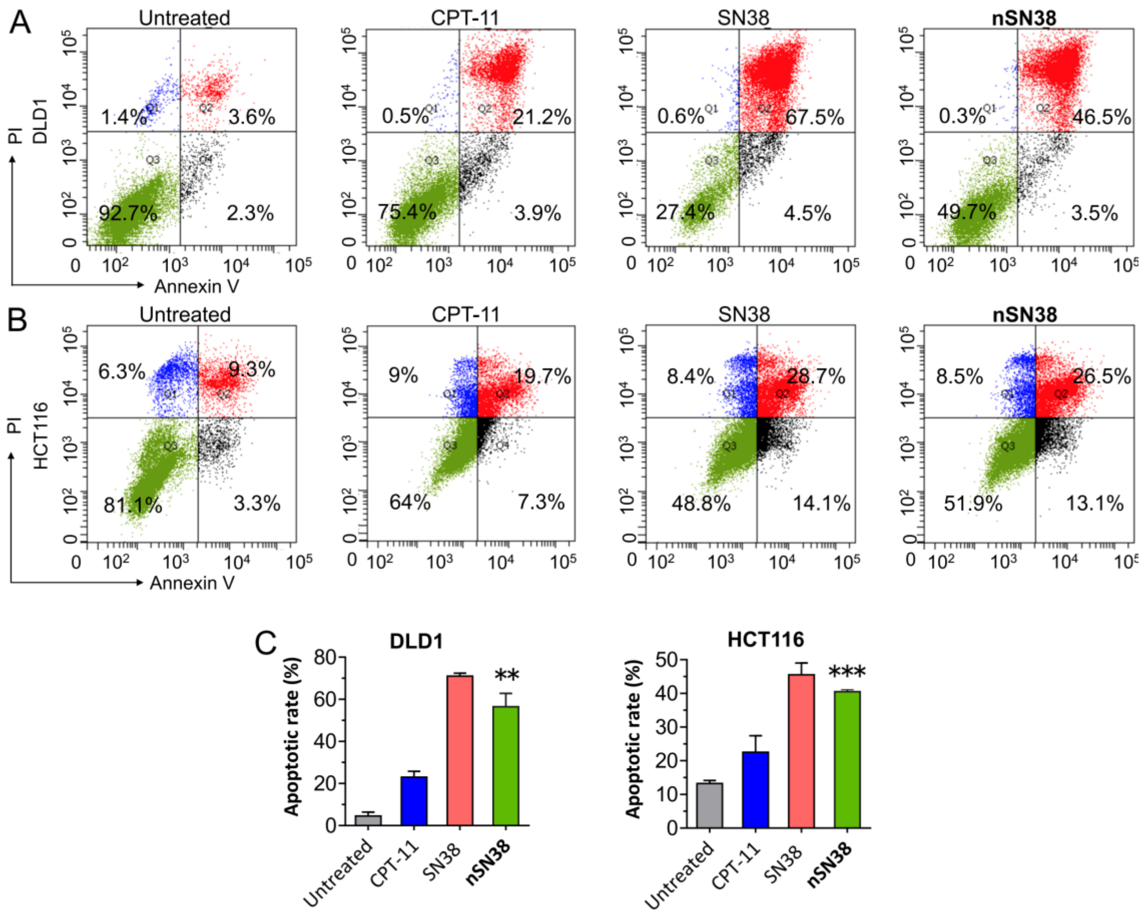

Figure S8. The *in vitro* cytotoxicity against colon cancer cell lines. Apoptotic analysis of DLD1 (**A**) and HCT116 (**B**) cells determined by FACS (Alexa Fluor 488 Annexin V/PI assay). (**C**) The percentage of apoptotic cells in each group is shown in the histogram. The cells were treated with the same concentrations (10 nM) of CPT-11, SN38, and **nSN38** for 48 h. Untreated cells were included as controls. The data are presented as the mean ± SD (n=3); ***p* < 0.01 and ****p* < 0.001 as compared to untreated cells.

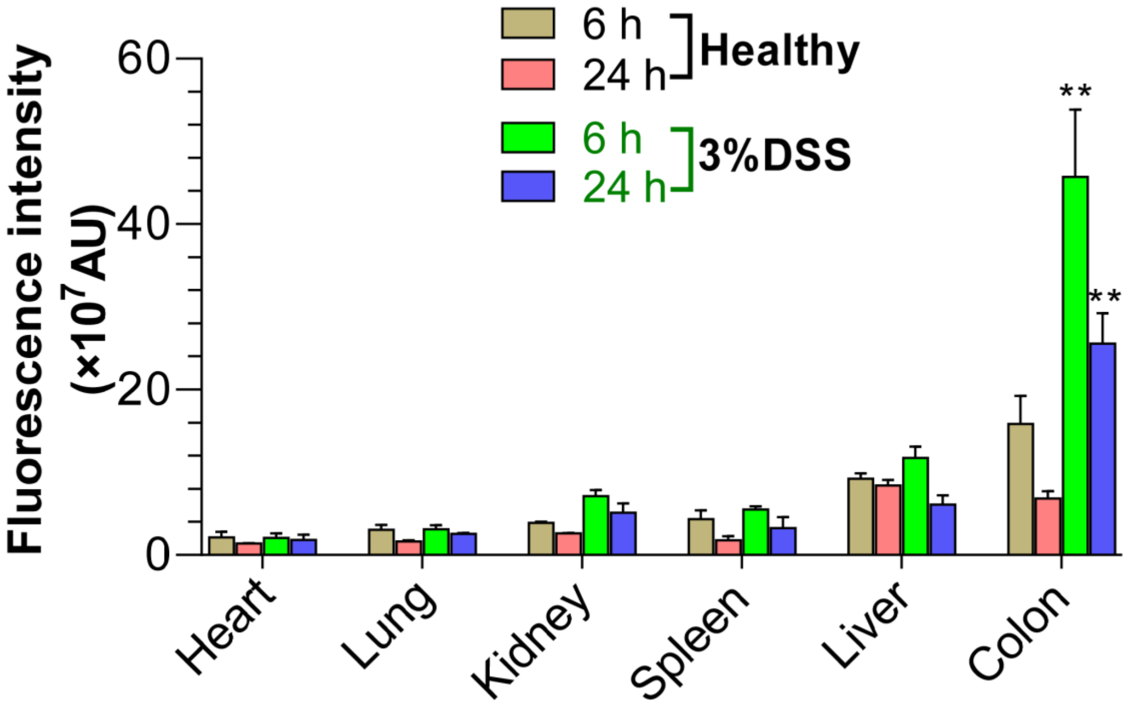

Figure S9. Quantitative analysis of fluorescence intensity of major organs excised at 6 or 24 h postadministration. The data are presented as the mean ± SD (n=3); ***p* < 0.01 as compared to the fluorescence intensity of the mouse colons in healthy mice.

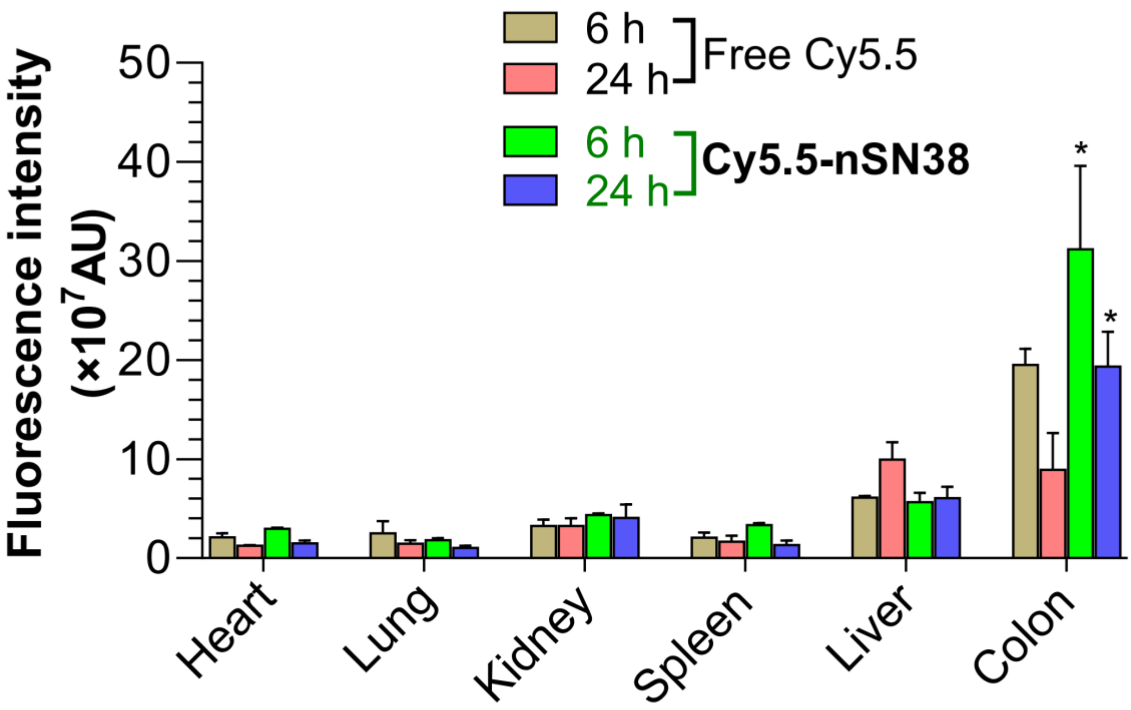

Figure S10. Quantitative analysis of fluorescence intensity of major organs excised at 6 or 24 h postadministration. The data are presented as the mean ± SD (n=3); *$p < 0.05$ as compared to fluorescence intensity of the mouse colons that were treated with free Cy5.5.

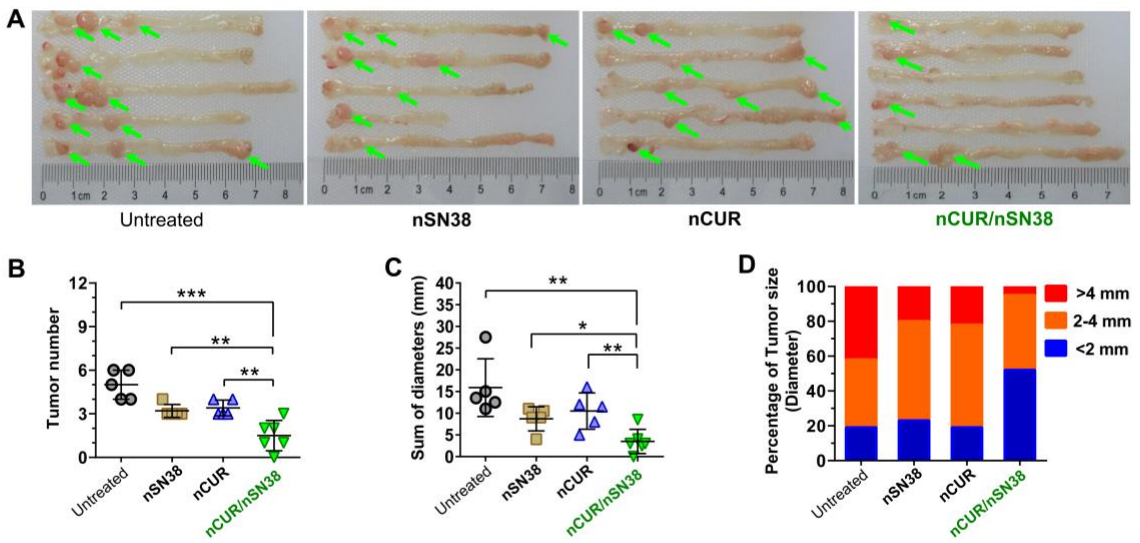

Figure S11. The synergistic effect of the combination of **nCUR/nSN38** nanotherapeutics on the tumorigenesis and the progression of CAC in a mouse model. (**A**) Representative images of murine colons. Tumor numbers per mouse (**B**), tumor diameters (**C**), and tumor size distribution (**D**) were measured in each group. The data are presented as the mean ± SD (n = 5 or 6). *$p < 0.05$, **$p < 0.01$, and ***$p < 0.001$.

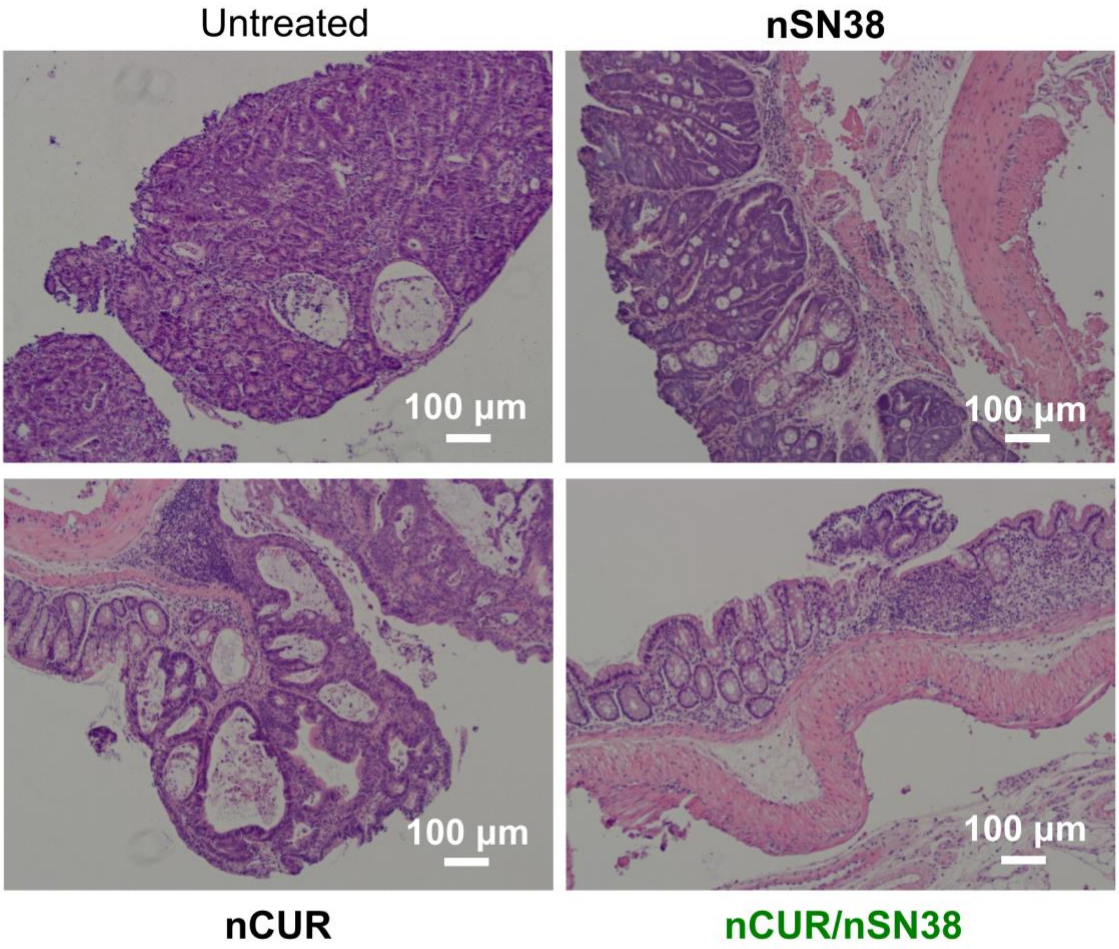

Figure S12. The synergistic effect of the combination of **nCUR/nSN38** nanotherapeutics on the tumorigenesis and the progression of CAC in a mouse model. H&E staining of tumor morphology.

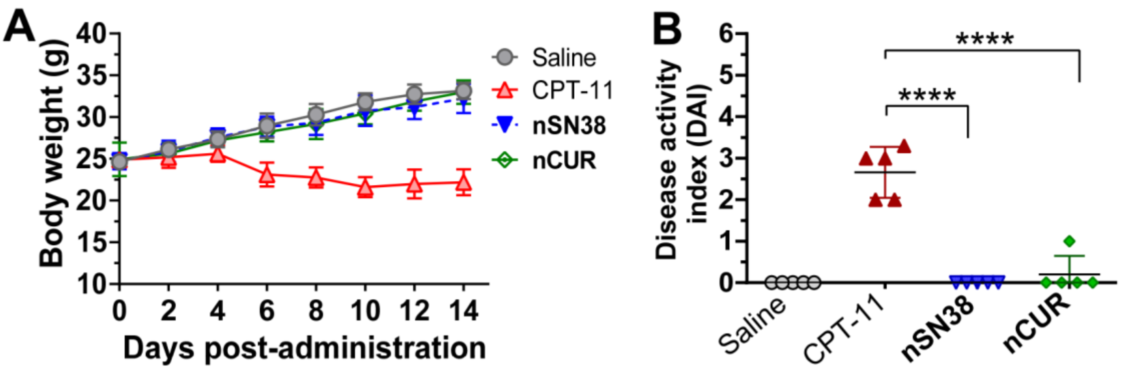

Figure S13. Toxicity evaluation of **nSN38** and **nCUR** as compared to CPT-11 in healthy ICR mice. (**A**) Changes in the body weights during the treatment period. (**B**) DAI was assessed on day 14 after administration of various drugs. The data are presented as the mean ± SD (n = 5); ****$p < 0.0001$. DAI is the summation of the stool consistency index, fecal bleeding index, and weight loss index.

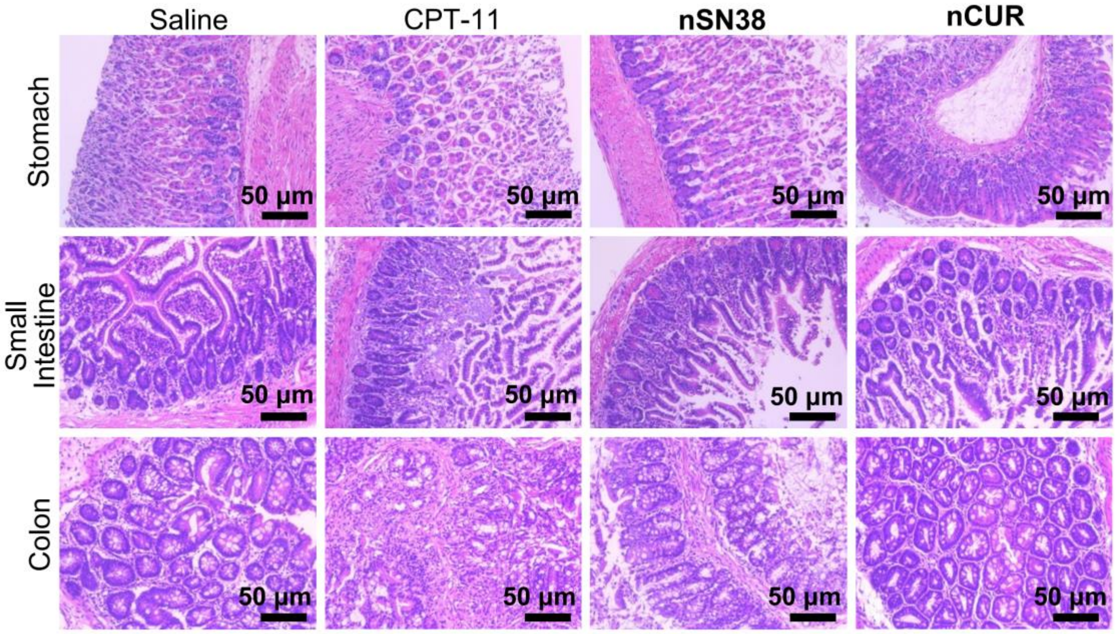

Figure S14. Toxicity evaluation of **nSN38** and **nCUR** as compared to CPT-11 in healthy ICR mice. Representative images of H&E staining of stomach, small intestine, and colon after drug treatment.

References:

[1] M.R.C. Marques, R. Loebenberg, M. Almukainzi, Simulated Biological Fluids with Possible Application in Dissolution Testing, Dissolut Technol, 18 (2011) 15-28.

[2] K. Livak, Relative quantification of gene expression: ABI Prism 7700 sequence detection system, Applied Biosystems User Bulletin, 2 (1997).

[3] K.J. Livak, T.D. Schmittgen, Analysis of relative gene expression data using real-time quantitative PCR and the 2(-Delta Delta C(T)) Method, Methods (San Diego, Calif.), 25 (2001) 402-408.
